# Attenuated processing of task‐irrelevant speech and other auditory stimuli: fMRI evidence from arithmetic tasks

**DOI:** 10.1111/ejn.16616

**Published:** 2024-11-25

**Authors:** Artturi Ylinen, Minna Hannula‐Sormunen, Jake McMullen, Erno Lehtinen, Patrik Wikman, Kimmo Alho

**Affiliations:** ^1^ Department of Psychology University of Helsinki Helsinki Finland; ^2^ Department of Teacher Education University of Turku Turku Finland; ^3^ Education Academy Vytautas Magnus University Kaunas Lithuania; ^4^ Advanced Magnetic Imaging Centre, Aalto NeuroImaging Aalto University Espoo Finland

**Keywords:** arithmetic, attention, cognitive control, distraction, speech

## Abstract

When performing cognitive tasks in noisy conditions, the brain needs to maintain task performance while additionally controlling the processing of task‐irrelevant and potentially distracting auditory stimuli. Previous research indicates that a fundamental mechanism by which this control is achieved is the attenuation of task‐irrelevant processing, especially in conditions with high task demands. However, it remains unclear whether the processing of complex naturalistic sounds can be modulated as easily as that of simpler ones. To address this issue, the present fMRI study examined whether activity related to task‐irrelevant meaningful speech is attenuated similarly as that related to meaningless control sounds (nonsense speech and noise‐vocoded, unintelligible sounds). The sounds were presented concurrently with three numerical tasks varying in difficulty: an easy control task requiring no calculation, a ‘routine’ arithmetic calculation task and a more demanding ‘creative’ arithmetic task, where solutions are generated to reach a given answer. Consistent with their differing difficulty, the tasks activated fronto‐parieto‐temporal regions parametrically (creative > routine > control). In bilateral auditory regions, activity related to the speech stimuli decreased as task demands increased. Importantly, however, the attenuation was more pronounced for meaningful than nonsense speech, demonstrating that distractor type can strongly modulate the extent of the attenuation. This also suggests that semantic processing may be especially susceptible to attenuation under conditions with increased task demands. Finally, as this is the first study to utilize the ‘creative’ arithmetic task, we conducted exploratory analyses to examine its potential in assessing neural processes involved in mathematical problem‐solving beyond routine arithmetic.

## Graphical abstract

In noisy environments, the attenuation of task‐irrelevant auditory processing is crucial for maintaining performance. Here, we demonstrate that the processing of task‐irrelevant meaningful speech can be disproportionately attenuated under high task demands, compared to meaningless control stimuli. This suggests that, at least in some conditions, the processing of ecologically relevant sounds with a high potential to distract can be regulated similarly as that of simpler ones.

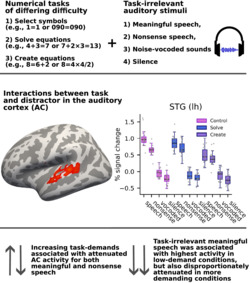

**Abbreviations**
AC: auditory cortex
AG: angular gyrus
ANOVA: analysis of variance
CG: cingulate gyrus
dlPFC: dorsolateral prefrontal cortex
DMN: default mode network
FD: framewise displacement
fMRI: functional magnetic resonance imaging
GLM: general linear model
IFG: inferior frontal gyrus
IPS: intraparietal sulcus
ITG: inferior temporal gyrus
LMM: linear mixed model
MFG: middle frontal gyrus
ROI: region of interest
SFG: superior frontal gyrus
SMG: supramarginal gyrus
STG: superior temporal gyrus
STS: superior temporal sulcus
vmPFC: ventromedial prefrontal cortex

## 1 INTRODUCTION

### 1.1 Theoretical background

‘Cognitive control’ is an umbrella term denoting the set of neurocognitive processes that organize our actions in a goal‐dependent manner (Diamond, 2013; Miller & Cohen, 2001). These processes are crucial not only in guiding overt behaviour but also in covert cognitive performance, where strategic information processing and maintaining attention to the current task are essential. In everyday situations, the demand for control during cognitive tasks is frequently increased by various distractors, such as background noises, that can capture our attention and interfere with task performance. Thus, cognitive control is often required simultaneously to guide task performance and to monitor and adapt to environmental contingencies (Badre, 2008; Gratton et al., 2018). Despite the frequency of such situations in everyday life, these dual aspects of cognitive control have not generally been studied simultaneously–that is, studies seldom manipulate both task demands (e.g., working memory load or other aspects of task difficulty) and distractor features (e.g., their complexity or salience) concurrently.

When focusing on cognitive tasks in conditions with task‐irrelevant distractors, the facilitation and suppression of task‐relevant and task‐irrelevant processes can interact in complex ways (Bidet‐Caulet et al., 2015; SanMiguel et al., 2010). On the one hand, increasing the demands of a cognitive task often leads to reduced processing of task‐irrelevant stimuli in sensory regions. Much of the research on this topic has been conducted in the context of the *Load Theory* (Lavie, 2005; Lavie & Tsal, 1994), which was originally formulated to resolve controversies relating to the locus of attentional selection (i.e., the early vs. late selection views; e.g., Broadbent, 1958; Deutsch & Deutsch, 1963). The basic tenet of *Load Theory* is that the locus of attentional selection is modulated flexibly, and that task‐irrelevant information is processed only when all attentional resources are not consumed by the task. A recent review on neuroimaging and electrophysiological studies related to *Load Theory* indicates that this idea is reasonably well supported by the existing evidence (Brockhoff et al., 2022). Relating to auditory distractors during cognitive tasks, in particular, studies have demonstrated that distractor‐related responses and auditory cortex (AC) activations are often decreased in conditions with increased task demands (Berti & Schröger, 2003; Ghatan et al., 1998; Marsh et al., 2015; Parmentier, 2014; Sörqvist et al., 2016).

While the reduction of sensory processing may come about as the consequence of depleted attentional resources, as emphasized by *Load Theory*, there is evidence suggesting that additional compensatory mechanisms may also be involved. Particularly, the sensory region deactivations have in many studies been coupled with increased activity in brain areas associated with aspects of cognitive control, such as the lateral‐fronto parietal network, which includes regions along the middle frontal gyrus (MFG) and in the posterior parietal cortex (Uddin et al., 2019). These activations have been suggested to reflect compensatory processes increasing the resources allocated to task processing, or the recruitment of control mechanisms in suppressing task‐irrelevant processing, or both (Ghatan et al., 1998; Gisselgård et al., 2004; Kulasingham et al., 2021; Schneider et al., 2021; Weissman et al., 2004). Taken together, these results demonstrate that the processing of task‐irrelevant and potentially distracting stimuli can be notably modulated by the cognitive demands of the task at hand.

On the other hand, features of the task‐irrelevant distractors, such as their saliency (Eltiti et al., 2005), also affect the extent to which they are processed, as well as behavioural performance in the task. In the case of auditory stimuli, louder, more variable, emotional and less predictable stimuli seem to have more potential for capturing attention and bringing about distraction (Alikadic & Röer, 2022; Hughes, 2014; Röer et al., 2017a; Sussman et al., 2003). This seems logical, given that the brain needs to continuously monitor environmental contingencies to detect and react to important events, and more salient stimuli often signal higher relevance. Moreover, these effects suggest that a comprehensive theory of load effects needs to consider the characteristics of distractor stimuli as an important determinant of the extent to which task‐irrelevant information is processed. Yet, most research on this topic has relied on relatively artificial and simplistic stimuli, raising the question of how well existing findings generalize to more naturalistic situations, in which one is often subjected to more complex and ecologically relevant stimuli.

In everyday life, speech is a particularly prominent and frequently encountered type of auditory stimulus. We spend significant amounts of time listening and attending to speech, thereby constantly training the neural networks involved in its processing (Addleman & Jiang, 2019). Therefore, it is not surprising that even unattended speech can induce semantic processing and activate language‐related networks of the brain (Har‐shai Yahav & Zion Golumbic, 2021; Rämä et al., 2012; Röer et al., 2017b). Some evidence also exists for meaningful as compared to meaningless speech being particularly potent in interfering with task performance, suggesting that the semantic content of speech may play a role in its ability to distract (Hughes & Marsh, 2020; LeCompte et al., 1997; Röer et al., 2019). However, this effect has not been robustly established, and it seems to depend on specific properties of the task and of the distractor (e.g., whether the task involves semantic processing, semantic relatedness of task and distractor stimuli and the signal‐to‐noise ratio of the irrelevant speech; Aydelott et al., 2015; Marsh et al., 2014). This variability may reflect the presence of compensatory mechanisms or load effects that typically suppress task‐irrelevant speech processing, but in some cases fail to do so when the stimulus is particularly salient or related to the task at hand. In any case, the neural processes underlying these effects remain underexplored.

Task‐related cognitive control involves largely domain‐general processes that are thought to aid in task performance by specifying and orchestrating the relevant sub‐operations in a flexible manner. These functions are associated with activity in a ‘task‐positive’ or ‘multiple demand’ network of primarily frontal and parietal brain regions, which typically show increased activity when any complex task is being performed (Duncan, 2010; Fedorenko et al., 2013). In any specific task, however, the domain‐general mechanisms are required to dynamically interact with domain‐specific systems (Cole et al., 2013; Woolgar et al., 2011). Mathematical tasks provide a prime example of a cognitive domain where performance depends on a well‐coordinated interplay of neurocognitive ‘core’ systems (for magnitude and quantity representation), and more domain‐general systems for sensory processing, memory and cognitive control (Dehaene et al., 2004; Henik et al., 2012; Iuculano et al., 2018). Mathematical tasks, in general, activate a fronto‐parieto‐temporal network of brain regions that partially overlaps with the lateral fronto‐parietal network mentioned above but also includes areas such as the angular gyrus (AG) and inferior temporal gyrus (ITG; Arsalidou & Taylor, 2011; Dehaene et al., 2004; Grotheer et al., 2018). Crucially, the involvement of different functional systems in mathematical processing is modulated depending on the nature of the task at hand. In arithmetic, for instance, frontal regions including the dorsolateral prefrontal cortex (dlPFC), inferior frontal gyrus (IFG) and cingulate gyrus (CG) seem to be mainly activated when a solution to a problem cannot be retrieved from memory, but requires procedural problem‐solving strategies (i.e., decomposing a calculation into smaller steps, choosing which strategy to adopt; Iuculano et al., 2018; Sokolowski, Hawes, & Ansari, 2023; Taillan et al., 2015). Beyond arithmetic, ‘higher’ mathematical processes also seem to rely largely on this fronto‐parieto‐temporal network (Amalric & Dehaene, 2016, 2019); however, there are studies suggesting that higher mathematical problem‐solving may also recruit systems involved in semantic processing to a greater extent than simple calculation (Liu et al., 2017, 2019; Zhou et al., 2018). This multiplicity and task dependency of the involved neurocognitive systems underscores the utility of mathematical tasks as models of complex problem‐solving processes even beyond numerical cognition. Additionally, since mathematical processing in everyday life often occurs amidst various distractions (e.g., noisy classrooms), mathematical tasks also provide a relevant framework for studying distractor‐related cognitive control during cognitive tasks.

### 1.2 Aims and hypotheses of the present study

The present study aimed to determine whether the neural processing of task‐irrelevant meaningful speech can be attenuated similarly as that of meaningless control stimuli during cognitive tasks that differ in their demands. To this end, we had participants perform numerical tasks of varying difficulty under concurrent but task‐irrelevant auditory stimulation while their brain activity was scanned using functional magnetic resonance imaging (fMRI). We employed four auditory conditions, which were designed with the goal of isolating effects related to naturalistic speech in particular: 1) Meaningful speech, consisting of conversations on emotionally neutral topics (e.g., hobbies) between two speakers, 2) nonsense speech, otherwise similar to the meaningful speech stimuli and spoken by the same speakers, but containing no meaningful words (cf. Moisala et al., 2015), 3) noise‐vocoded sounds that were generated based on the speech stimuli and thus controlled for certain physical aspects of the signal, but were unintelligible and did not sound like speech (Shannon et al., 1995) and 4) silence (except for MRI scanner sounds). We expected that increasing task demands would lead to reduced distractor‐related activity in auditory regions and aimed to examine whether activity related to meaningful speech could be attenuated in a manner comparable to that of the other stimuli. Additionally, given earlier research demonstrating fronto‐parietal activations in conditions with distraction (discussed above), we expected the meaningful speech distractor to be associated with an increased fronto‐parietal activity.

As cognitive tasks, we employed three numerical tasks differing in their demands: 1) an easy control task requiring no calculation, 2) a ‘routine’ arithmetic calculation task, 3) a more demanding ‘creative’ arithmetic task, where the instruction is to create equations adding up to a given target number. Like routine calculation, the creative task requires numerical processing and calculation, but it also imposes additional demands for multiple aspects of cognitive control required in complex problem solving, such as for working memory in manipulating numbers and interim results, and cognitive flexibility in finding new solutions. Thus, these tasks are relatively realistic in that they simulate everyday problem‐solving scenarios requiring both calculation and higher‐order cognitive skills, therefore serving as a relevant framework also for studying higher cognitive processing under distraction. We hypothesized that as task difficulty increases, there would be progressively greater engagement of the ‘task‐positive’ networks, including the lateral fronto‐parietal network discussed above, which are typically more active during cognitively demanding tasks compared to less demanding control conditions (Fedorenko et al., 2013; Fox et al., 2005).

The creative arithmetic task was originally developed to assess school‐aged children's ‘adaptive number knowledge’, including knowledge of numbers, their interrelations (e.g., multiples, factors, exact and approximate relations), arithmetic operations and their flexible use (Lehtinen et al., 2015; McMullen et al., 2016). Research has shown significant individual variability in performance in this task, evident not only in the quantity of arithmetic expressions generated but also in their complexity (McMullen et al., 2016, 2019; Pehkonen et al., 2022). Therefore, the task might prove useful also in shedding light on the neural basis of mathematical problem‐solving beyond rote calculation, as well as individual differences therein. Thus, the secondary goal of the present study was to perform exploratory analyses on brain activations during creative vs. routine arithmetic performance. Because the two arithmetic tasks differ in several aspects, contrasting them cannot be used to pinpoint the neural activations behind any single task‐related cognitive process. We therefore focused particularly on interactions between task and calculation complexity (simple vs. complex equations, i.e., with one or two arithmetic operators, respectively): while increases in task demands and calculation complexity are both expected to recruit ‘task‐positive’ networks (Fedorenko et al., 2013; Fox et al., 2005), interactions between these factors can reveal processes that are independent of mere difficulty and more specific to the task at hand. As the creative task has not been studied with neuroimaging methods before, we had no strong a priori hypotheses regarding these interactions.

## 2 MATERIALS AND METHODS

### 2.1 Participants

Twenty young adult participants (11 females, age 19–24 years, mean 22.4 years) were recruited from the University of Helsinki student mailing lists. The sample size was chosen without prior power analyses but was determined before starting the measurements drawing upon previous fMRI studies examining within‐subjects effects of distraction and mathematical tasks (e.g., Smucny et al., 2013; Zhou et al., 2018). Inclusion and exclusion criteria were established prior to the measurements and required all participants to be healthy, right‐handed native speakers of Finnish, with self‐reported normal hearing and normal or corrected‐to‐normal vision and no self‐reported history of neurological or psychiatric disorders. Participants were screened for these inclusion criteria during a phone interview prior to the measurement and through questionnaires completed on‐site.

### 2.2 Ethics

All participants gave written informed consent and were monetarily compensated for their participation (15 €/h). Their suitability for fMRI scanning was screened following standard procedures of the imaging facility, the Advanced Magnetic Imaging Centre at Aalto University, Espoo, Finland. The experiment was conducted in accordance with the Declaration of Helsinki and the experimental protocol was approved by the Ethics Committee of the Hospital District of Helsinki and Uusimaa, Finland.

### 2.3 Tasks and calculation types

**FIGURE 1:**
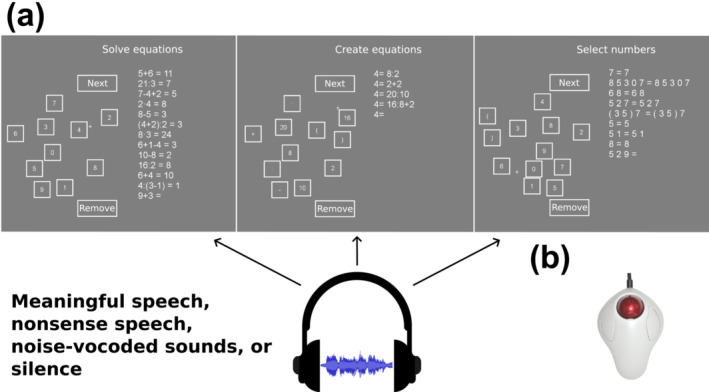
A) The present experiment consisted of three numerical tasks, which were factorially combined with four types of concurrent auditory background stimuli. In the numerical tasks, participants either solved (left) or created (middle) arithmetic equations or selected symbols to match shown ones (right). As for the auditory stimuli, meaningful speech comprised conversations on emotionally neutral everyday topics (e.g., hobbies) spoken by two speakers. Nonsense speech was otherwise similar to meaningful speech but contained no meaningful words. Noise‐vocoding (Shannon et al., 1995) with three logarithmically spaced frequency bands was used to create an unintelligible stimulus type that nonetheless controls for certain physical aspects of the speech signal. In the silent condition, no auditory distractor was presented (scanner sounds were present in all conditions). The tasks were projected on a mirror mounted on the head coil in front of the participant, while the auditory stimuli were presented through noise‐cancelling earbuds. B) The tasks were performed using an MRI‐compatible trackball mouse to control a cursor (small + in the figures) and select symbols on the screen by clicking them.

The experiment included three numerical tasks (Figure 1A), which were factorially combined with different levels of concurrent auditory stimulation (described in detail in section 2.4), and were performed in 60 s long experimental blocks during which the task and distractor types remained constant. All three tasks were performed using an MRI‐compatible trackball mouse (Figure 1B) to move a cursor and to click on symbols on the screen in a self‐paced manner. Self‐pacing was necessary due to the nature of the creative arithmetic task that required the participants to be free to form any expression they wished based on the available numbers and arithmetic operators (please see below for details).

In the routine calculation or ‘solve’ task, participants were to perform arithmetic calculations (Figure 1 A, left). Equations in this task included both simple two‐operand calculations (e.g., ‘3+4’ or ‘10:5’) that are considered likely to be solved using a retrieval strategy, and complex calculations with three operands (e.g., ‘5+1×3’), that are considered likely to prompt procedural problem‐solving strategies (Sokolowski, Hawes, & Ansari, 2023). All four basic arithmetic operations were used in the calculations. Simple additions, subtractions, multiplications, divisions and complex calculations each accounted for 20% of all calculations. In all additions, both operands were numbers between 0 and 10; in all subtractions, all differences were between 0 and 10; in all multiplications, both operands were single‐digit numbers; in all divisions, the solution was between 1 and 10. Complex equations included three single‐digit operands, with half of them also involving parentheses. All operands and solutions to all equations were positive whole numbers.

In the creative arithmetic task, or the ‘create’ task, participants were given a target answer and a set of five numbers and arithmetic operators from which they were to construct equations that add up to the given answer (Figure 1 A, middle). For instance, if the target answer is ‘8’, and ‘2’, ‘4’, ‘6’, ‘16’ and ‘24’ are given as the set to operate with, two possible answers could be ‘6+2’ and ‘4×4/2’. The sets of target answers and given numbers were chosen so that there were several easily identifiable arithmetical relations between them and, therefore, also a large number of potential solutions to the task (for previous research on this task, see McMullen et al., 2016, 2017, 2019). To maximize the creation of unique solutions to the task at hand, the participants were asked to refrain from providing addition and multiplication solutions that only differed in the order of operands (e.g., ‘2+6’ and ‘6+2’). In analyses, similarly as in the solve task, the created equations were classified as simple if they comprised one operator, and complex otherwise. Note, however, that in the creative task, participants could form more complex equations than any calculation in the solve task (i.e., more than two operators); therefore, to avoid the confounding effects this might produce, these most complex calculations were not included in the analysis on interactions between task and complexity (for details, please refer to section 2.8.1).

In the control task, participants were presented with a string of symbols, and their task was simply to select corresponding symbols to match the given string (Figure 1 A, right). Expressions consisted of 1, 2, 3 or 5 numbers (e.g., ‘2 4 8 7 9’), or of 3 numbers and parentheses (e.g., ‘8 (3 2)’). These expression types were chosen so that visual stimulation (i.e., seeing numerical expressions) and cursor movements during the control task would match the other tasks as well as possible. Lists of all calculations used in the solve task, sets of target answers and given numbers in the create task and expressions in the control task can be found in Tables S1, S2 and S3, respectively, in the Supplementary Materials.

In the experimental interface, the current and previously solved problems were located on one side of the screen, while the possible inputs (i.e., numbers and operators) were located on the other side (Figure 1A). The left–right locations of these two elements were randomly assigned at the start of each block. As participants inputted their answers by clicking on the white boxes, the input appeared on the other side of the screen to the current equation or expression. In the create task, the set of available numbers varied between task blocks. This required the participants to visually scan through the available numbers, at least at the start of each block. To control for this across the tasks, the numbers were presented in a scrambled manner also in the other tasks. The configuration of the white boxes remained identical from block to block within a task, but the numbers were placed in them randomly in each block. The experimental interface also included a button to move on to the next calculation after the previous one was completed (‘Next’) and a button to erase the current calculation in case an error was identified (‘Remove’; Figure 1A). After clicking the ‘Next’ button, the next calculation appeared below the previously completed one, whereas after clicking the ‘Remove’ button, the input given to the current calculation disappeared.

Because the participants performed the calculations in a self‐paced manner, the number of solutions per block varied. To ensure that all participants solve all calculation types in the solve blocks, the six first calculations in these blocks always included one simple calculation with each of the four basic arithmetic operations, as well as two complex calculations, one with and one without parentheses. Starting from the seventh calculation, the different calculation types were presented in a randomized order. Similarly, in each block of the control task, the first five calculations always consisted of expressions with one number, two numbers, three numbers, five numbers and three numbers with parentheses, after which the different expression types were presented in a randomized order. For each block of the solve and control tasks, the experiment included 20 different calculations or expressions, respectively (see Tables S1 and S3 in the Supplementary Materials). Had a participant reached 20 calculations in a single block, the experimental interface would have repeated the same calculations from the start; however, no participant reached 20 calculations in any block.

### 2.4 Auditory distractors

The tasks were factorially combined with four auditory distractor conditions: 1) meaningful speech, 2) nonsense speech, 3) noise‐vocoded sounds and 4) silence (except for scanner sounds). Because we utilized the meaningful and noise‐vocoded distractors also in another study (in preparation) with school‐aged participants, the meaningful speech distractors were designed to mimic conversations that could be heard in a classroom and classroom babble was also added to the background of the conversations (both in the meaningful and nonsense speech conversations, as well as in the noise‐vocoded distractors; see below for details).

The meaningful speech distractors consisted of conversations on emotionally neutral everyday topics, such as hobbies and school. To control for phonological content, the nonsense conversations were created based on originally meaningful ones by randomly shuffling the vowels within a conversation. The conversations were then checked by a research assistant trained in linguistics and edited to ensure that each nonsense word followed the phonotactic rules of Finnish (e.g., which diphthongs are allowed in Finnish). Constructed in this manner, the nonsense conversations were as similar as possible to the meaningful ones, but with no meaningful words. To ensure that the relationship between the meaningful and nonsense conversations remained opaque to the participants, we did not record the originally meaningful scripts that were used to create the nonsense scripts.

The conversations were recorded in a soundproof studio with two speakers, one male and one female (both aged 12). Each conversation consisted of 12 lines. To achieve natural speech rhythm and intonation in the nonsense sentences, the speakers first read out loud the meaningful sentence based on which the nonsense sentence was created and immediately afterwards uttered the nonsense sentence as similarly as possible. This procedure was repeated as many times as necessary to achieve a natural‐sounding utterance. The meaningful sentences were recorded in a similar fashion, one at a time and repeated if necessary. In editing, the recorded sentences were then combined to form entire conversations, each lasting 62 seconds (mean sentence length in meaningful and nonsense speech was 4.34 and 4.36 seconds, respectively). Subsequently, a recording of classroom babble was added to the conversations. These background tracks consisted of classroom sounds such as babble, although with no understandable speech. The background tracks were taken from a recording of classroom sounds that is distributed under the Creative Commons license (https://freesound.org/people/reinsamba/sounds/73178/), from which we cut 62 s long excerpts that contained no discernible words or other salient sound events. While there were brief pauses in between the lines spoken by the speakers, the addition of the classroom sounds ensures that the distractor soundtracks provide continuous auditory stimulation during the whole length of each task block. The volume of the classroom sounds was set at approximately 3 dB below the volume of the conversations. Finally, the combined tracks were low pass filtered using a cutoff of 5000 Hz and normalized to the same peak sound energy. We used Adobe Audition CS6 (Adobe Systems Inc., San Jose, CA, USA) for audio editing.

Noise‐vocoding (Shannon et al., 1995) was used to create a type of auditory distraction that does not sound like speech but still controls for certain physical aspects of the speech signal by preserving temporal cues while degrading spectral information. In noise‐vocoding, amplitude envelopes are extracted from logarithmically divided frequency bands of a speech stream and used to modulate white noise. Using this technique, one can vary the intelligibility of the noise‐vocoded speech, which depends on the number of bands used (Davis & Johnsrude, 2003). Here, we used only three bands (cutoffs at 50, 247, 1216 and 6000 Hz) in noise‐vocoding the previously described soundtracks with conversation and classroom babble, resulting in completely unintelligible sounds. Noise‐vocoding was performed using Praat (version 6.027; Boersma & Weenink, 2001), and the noise‐vocoded tracks were normalized to the same peak sound energy as the meaningful and nonsense speech distractors.

### 2.5 Procedure

Before the fMRI session, all participants underwent a practice session during which they were familiarized with the experimental interface, using the trackball mouse and the different tasks. The participants were asked to perform the tasks as quickly and accurately as possible and to ignore any sounds they heard during the tasks. Additionally, when performing the tasks, the participants were instructed to first decide on the answer that they will input next, and only then move the cursor.

The fMRI experiment consisted of four runs each including 12 blocks, comprising one presentation of each of the three tasks combined with each of the four auditory distractors. The duration of the task blocks was 62 s, beginning with a 2 s long instruction period followed by 60 s of task performance. During the instruction period, an instruction on which task was to be performed next could be seen in the top part of the experimental interface (Figure 1A), and the white boxes were already present on the other side of the screen, but the numbers and operators in the boxes were masked with ‘X'‐symbols. After each block, a feedback message with the sentence ‘Well done!’ was presented for 2 s. There was a rest period of 10 s between each block and an additional rest period of 40 s after the sixth block. The duration of one fMRI run was thus ca. 15 min. The order of blocks within a run was randomized, and the order of runs was counterbalanced between participants.

We used Presentation 20.0 (Neurobehavioral Systems, Berkeley, CA, USA) to control the experiment. The experimental interface was projected onto a mirror mounted on the head coil. The participants performed the tasks using an MRI‐compatible trackball mouse (HHSC‐TRK‐1, Current Designs, Philadelphia, PA, USA). Sound stimuli were presented binaurally through earphones including canal tips that also acted as earplugs (Sensimetrics Model S14; Sensimetrics, Malden, MA, USA). The sound intensity was determined individually to be pleasant but loud enough to be heard among scanner noise (approximately 80 dB SPL at the eardrum). Scanner noise (ca. 102 dB SPL, as measured in the head coil) was attenuated by the earplugs and with viscoelastic mattresses around and under the head of the participant inside the coil. Participants' eye movements were tracked using an EyeLink 1000 eye tracker (SR Research, Mississauga, Ontario, Canada). Due to technical difficulties, however, this data was not usable for several participants and, thus, we decided not to analyse the eye movements further in the present study.

### 2.6 (f)MRI data acquisition and preprocessing

Magnetic resonance imaging was performed at the Advanced Magnetic Imaging (AMI) Centre (Aalto Neuroimaging, Aalto University School of Science, Espoo, Finland) using a 3 Tesla Magnetom Skyra whole body scanner (Siemens Healthcare, Erlangen, Germany). We used a 30‐channel head coil, modified from a 32‐channel coil to enlarge the visual field by removing the parts in front of the eyes. The experiment included four functional runs of 738 volumes, consisting of 56 oblique axial slices of T2*‐weighted simultaneous multislice (multiband factor 4) echo‐planar images (TR: 1300 ms, voxel matrix 96 × 96, in‐plane resolution: 2.5 mm isotropic, TE: 41 ms, flip angle: 65°). After the last functional run, a high‐resolution anatomical image was acquired (MPRAGE sequence, voxel matrix: 176 × 256 × 256, in‐plane resolution: 1 mm isotropic, TR: 2530 ms, TE: 3.3 ms).

We used a standard preprocessing pipeline implemented in *fMRIPrep* 20.2.5 (Esteban et al., 2018) to preprocess the functional and structural MRI data. Please see the Supplementary Materials for a detailed description of the preprocessing steps generated by *fMRIPrep*.

### 2.7 Behavioural data analysis

Behavioural performance was measured as the average number of correct answers per block. We tested whether behavioural performance was affected by the different auditory conditions using a 3 × 4 repeated measures analysis of variance (ANOVA) with factors Task (solve, create, control) and Distractor (speech, nonsense, vocoded, silence). Note that because the number of symbols to be inputted per calculation or expression varied between the tasks (i.e., expressions to be inputted in the control and creative arithmetic tasks were on average longer than solutions in the solve task), the main effect of Task is not highly informative on performance. To assess whether auditory distractor type affected simple and complex calculations differently and whether their distribution differed between the create and solve tasks, we analysed the percentage of correctly performed complex calculations out of all correctly performed calculations using a 2 × 4 repeated‐measures ANOVA with factors Task (solve, create) and distractor (speech, nonsense, vocoded, silence). As a measure of effect size, we used partial eta squared (η_p_^2^). Where Mauchly's test of sphericity indicated significant differences in variance between the factors in these ANOVAs, we report Greenhouse–Geisser corrected *P*‐values along with the correction value ε, but with the original degrees of freedom. The behavioural analyses were performed using jamovi version 2.4.11 (The jamovi project, 2022).

### 2.8 fMRI analysis

#### 2.8.1 First‐level analyses

We used FEAT (FMRI Expert Analysis Tool, version 6.00), part of FSL (FMRIB's Software Library, www.fmrib.ox.ac.uk/fsl), to perform four separate first‐level general linear model (GLM) analyses. The first GLM assessed changes in brain activity related to the different task and distractor conditions, irrespective of calculation complexity within the task. This model included event‐related regressors for each combination of task and distractor (12 in total), as well as regressors for task instructions, feedback and the temporal derivatives of these regressors. For all task and distractor conditions, the regressors modelled the time points of correctly performed calculations starting at the moment the problem appeared and stopping the moment the participant clicked the ‘Next’ button. FSL's custom three‐column format was used in modelling the events. In the create task, participants were able to create more complex calculations than were included in the solve task (i.e., calculations with three or more operators, whereas two operators were the maximum in the solve task). To avoid the confounding effects this may introduce when contrasting the two tasks, these calculations were assigned to a separate regressor of no interest; note that the most complex create task calculations were included in the fourth GLM described below.

In the first GLM, calculations were collapsed across simple and complex ones because not all participants created complex answers in all distractor conditions during the create task. Therefore, we did not conduct whole‐brain analyses modelling activity separately for each combination of task, distractor and calculation complexity. However, we wanted to examine activations partitioned by all these factors in a region of interest (ROI) analysis to determine whether calculation complexity affected the auditory cortex interactions observed through the first GLM described above. To this end, a second GLM was formed, modelling activity during simple and complex calculations separately for each task and distractor condition. Here, calculations in the solve and create tasks were classified as simple if they contained one arithmetic operator, and complex if they contained two arithmetic operators. The most complex answers were again excluded to avoid their potential confounding effects. Expressions in the control task were divided into short (one or two symbols) and long (more than two symbols) ones. Only correctly performed calculations in each task were included.

The third and fourth GLMs were performed to explore the effects of calculation complexity and interactions between task and calculation complexity in whole‐brain analyses. To maximize statistical power, these analyses were collapsed across all distractor conditions. For the third GLM, we again formed event‐related regressors and their temporal derivatives corresponding to the time points of correctly performed simple and complex calculations in the create and solve tasks. To allow for the comparison of complexity‐related effects in the solve and create tasks with effects related to expression length in the control task, short and long expressions in the control task were again modelled. The classifications for simple/complex and short/long were identical to the ones described above. Calculations with three or more operators in the create task were again not included. To minimize the effects of distractor‐related activity on the results of this analysis, the GLM included regressors coding for each of the four distractor conditions across all tasks (i.e., 4 distractor‐related regressors in total).

The final GLM was performed to further assess associations between brain activity and calculation complexity in the create task, this time also including the most complex create task calculations (i.e., with three or more operators). To this end, we formed a regressor coding for each correctly performed calculation in the create task with the number of operators that calculation contained. Thus, this regressor captures effects in regions where activity correlates with increasing calculation complexity. To control for activity related merely to inputting longer answers, associations between brain activity and expression length in the control task were also modelled, as each correctly inputted control expression was modelled with the number of symbols it contained. These regressors were demeaned before including them in the GLM. The solve task was omitted from this analysis because complexity‐related effects in that task were already assessed with the third GLM described above (and since it only contains calculations with one or two operators, all events were already included there). Regressors coding for each of the four distractor conditions were again included.

In all GLMs, incorrectly performed calculations, as well as those during which the participant used the ‘Remove’ button, were assigned to a separate regressor of no interest. To account for motion, drift and other nuisance factors, all GLMs included the following confound regressors calculated by *fMRIPrep*: global signal, the first five *aCompCor* regressors explaining most variance, framewise displacement (FD), the six basic motion parameters along with their second powers and derivatives and the discrete cosine basis functions. Regressors were also included to censor volumes with an FD value > 0.9 (Siegel et al., 2014). Finally, we included a regressor and its temporal derivative to account for brain activity related to moving the cursor. This regressor modelled all time points during which the participant moved the cursor (with a temporal resolution of 50 ms) with a value of 1, and all other time points with a 0. Regressors related to the experimental conditions, instructions, feedbacks and cursor movements were convolved with FSL's double‐gamma function (phase: 0). A smoothing of 5 mm full‐width half maximum was applied in FEAT in all GLMs.

#### 2.8.2 Group‐level whole‐brain analyses

Group‐level whole‐brain analyses were conducted for the first, third and fourth GLM described above (the second GLM was used only for ROI analyses). First‐level GLM results from the four runs of each participant were combined using FEAT's fixed effects model to obtain participant‐level results. In cases where a participant had a run with blocks containing no calculations acceptable as per the criteria outlined in the previous section, the participant‐level results were formed based on the three runs with acceptable calculations (this affected three participants in the first GLM, one participant in the third GLM and no participants in the fourth GLM; no participant had missing events in any condition in two or more runs). The participant‐level results were subsequently used as input to group‐level analyses. At the group level, whole‐brain ANOVAs were performed using the Multivariate and Repeated Measures toolbox (version 1.0; McFarquhar et al., 2016) with MATLAB R2018b (Mathworks Inc., Natick, MA, USA).

First, to examine differences in activation levels during the different tasks but irrespective of calculation complexity, we performed a 3 × 4 repeated measures ANOVA with factors Task (solve, create, control) and Distractor (speech, nonsense, vocoded, silence). Participant‐level contrasts from the first GLM described above were used as input.

Second, to assess effects related to calculation complexity in the solve and create tasks, we performed a 2 × 2 repeated measures ANOVA with factors Task (solve, create) and Calculation complexity (simple, complex). Participant‐level contrasts from the third GLM described above were used as input. The main effects of the Task from this ANOVA were left unanalysed, as they are already assessed in the previously mentioned analysis.

To assess associations between brain activity and calculation complexity in the create task including also the most complex calculations, a one‐way ANOVA was performed to contrast the regressors for associations between brain activity and calculation complexity in the create task, and brain activity and expression length in the control task (fourth GLM described above).

In all ANOVAs, family‐wise error rate (FWER) correction was applied to correct for multiple comparisons using cluster‐level permutation inference (5000 permutations). In the analysis of the main effects of Task, which revealed very large clusters in widespread areas of the brain, a stricter initial cluster forming threshold of *P* = 0.0001 was applied; in other ANOVAs, an initial cluster forming threshold of *P* = 0.001 was used. In all ANOVAs, cluster‐level results were thresholded at *P* = 0.05, and clusters smaller than 50 mm^3^ were discarded.

Because *F*‐values from an ANOVA provide no information on the direction of the effect, we used simple contrasts to mask the result images to illustrate what drives the significant results. The contrasts were calculated in FEAT for all possible within‐factor pairwise comparisons (for instance, to illustrate what drives significant main effects of Task, we calculated the following contrasts: create vs. solve, create vs. control, solve vs. control). A threshold of *z* = 1.96 was applied to the contrast images at this point. Subsequently, the contrasts images were combined to illustrate different combinations of effects. For the main effect of Task, we illustrate regions with a parametric task‐dependent modulation (create > solve > control); regions where either of the arithmetical tasks was associated with the highest activity, but no difference was observed between the other arithmetical task and the control condition, that is, create > (solve = control), and solve > (create = control); and regions where the control task was associated with highest activity, regardless of other possible differences (Figure 3). For the main effect of Distractor, we illustrate regions where the meaningful and nonsense speech distractors were both associated with higher activity than the other two conditions; regions where the speech distractor was associated with higher activity than any of the other distractor conditions; and regions where all other distractors were associated with higher activity than the speech distractor (Figure 4). Note that the ANOVA results are statistically corrected for multiple comparisons as described above, and the uncorrected *z‐*contrasts were used only for illustrating the direction of the effects.

For the main effects of Calculation complexity and interactions between Task and Calculation complexity in the exploratory analyses, we illustrate the direction of effects based on the plots in Figures S2 and S3 in the Supplementary Materials (i.e., simple > complex or vice versa for the main effects, and solve simple > solve complex and create complex > create simple or vice versa for interactions). The detailed plots of these effects are presented in the Supplementary Materials to maintain the main text as concise as possible. For the analysis of the associations between brain activity and calculation complexity (fourth GLM), we illustrate separately regions in which the effect was driven by a positive association during the create task, or a positive association during the control task (Figure 7, bottom).

For visualization, the result images from the group‐level analyses were projected onto the Freesurfer average surface (fsaverage) using Freesurfer's *mri_vol2surf* with a surface smoothing of 3 mm FWHM. Details on all observed clusters, including cerebellar and deep brain activations that are poorly or not at all visible on the surface renderings, are provided in Tables S5–S10 in Supplementary Materials.

#### 2.8.3 ROI analyses

Percent signal change values were extracted from ROIs to illustrate 1) what drove the significant interactions in the 3 × 4 whole‐brain ANOVA with factors Task and Distractor 2) how calculation complexity affected the AC activations observed in the aforementioned interactions, 3) what drove the speech‐related deactivations observed in fronto‐parietal regions in the analysis on the main effects of Distractor, 4) and to illustrate the direction of effects in the main effects of Calculation complexity and the interaction between Task and Calculation complexity.

As they were integral to the topic of the present study, the ROI values extracted from the AC clusters observed on interactions between Task and Distractor were subjected to posthoc tests, Bonferroni corrected across comparisons within each cluster to control for Type I errors. These examined whether activity during each distractor condition differed from each other between each task pair (e.g., meaningful speech during the control task from meaningful speech during the solve task), and whether the meaningful speech condition differed from the nonsense speech condition in each task.

Additionally, we examined whether activations in these AC clusters displayed the main effects of calculation complexity or interactions between Task, Distractor and Calculation complexity. Because some participants did not create complex calculations in all distractor conditions (in total, seven subjects had one missing event type, but none had more than that), we used linear mixed models (LMMs), to analyse these data, as LMMSs are better suited to handle missing data than repeated measures ANOVAs. The LMMs were fitted using restricted maximum likelihood estimation, and Satterwaithe's method was used to approximate the degrees of freedom. The models included a random intercept for participants, and random slopes for all repeated measures effects except for the highest‐order interaction. These analyses were performed using jamovi version 2.4.11 and the GAMLj module (Gallucci, 2019; The jamovi project, 2022). As these models were formed primarily to analyse the main effects of Calculation complexity and interactions between Task, Calculation complexity and Distractor, we only report these effects.

Note that to limit the number of statistical comparisons, post‐hoc tests were not conducted on ROI data on the speech‐related fronto‐parietal deactivations, nor on the data from clusters observed in the analyses on main effects of calculation complexity and interactions between Task and Calculation complexity.

## 3 RESULTS

### 3.1 Behavioural performance

**FIGURE 2:**
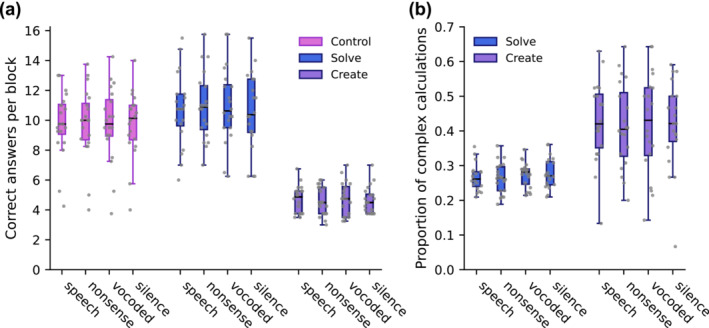
Behavioural performance was measured as the number of correctly performed trials in each task and distractor condition (per block, averaged over the four runs). Grey dots depict individual data points. A) Performance was not significantly affected by distractor type in any of the tasks. Note that although a main effect of task on the number of completed trials was observed, this effect is not of primary interest because entering solutions required more time on average in the control and create tasks than in the solve task (see main text for details). B) The distribution of correctly performed simple and complex trials in the solve and create tasks was not affected by distractor type, indicating that the distractor sounds did not differentially affect simple and complex calculations in either of the tasks. However, the participants completed proportionally more complex calculations in the create task.

Behavioural performance, measured as the number of correctly performed trials in each task and auditory distraction condition, is shown in Figure 2. A 3 × 4 repeated measures ANOVA revealed no significant main effect of Distractor (*F*_3,57_ = 0.54, *P* = 0.66, *η*_*p*_^*2*^ = 0.027), nor Task × Distractor interactions (*F*_6,114_ = 0.72, *P* = 0.58, *ε* = 0.64, *η*_*p*_^*2*^ = 0.036) on performance. While the main effect of Task was significant, it is not of primary interest, because the control and create tasks required the participants to input answers that were on average longer than answers in the solve task, thus taking more time (*F*_2,38_ = 162.01, *P* < 0.001, *η*_*p*_^*2*^ = .90). The distribution of simple and complex calculations was not significantly affected by the distractor type (main effect of Distractor: *F*_*3,57*_ = 0.18, *P* = 0.91, *η*_*p*_^*2*^ = 0.009) nor was there an interaction of task and distractor (*F*_*3,57*_ = 0.38, *P* = 0.77, *η*_*p*_^*2*^ = 0.019). However, the distribution of simple and complex calculations differed between the two arithmetic tasks (*F*_*1,19*_ = 22.77, *P* < 0.001, *η*_*p*_^*2*^ = 0.55), as there were proportionally more complex calculations in the create task.

### 3.2 fMRI results

#### 3.2.1 Main effects and interactions of task and auditory distractor type

**FIGURE 3:**
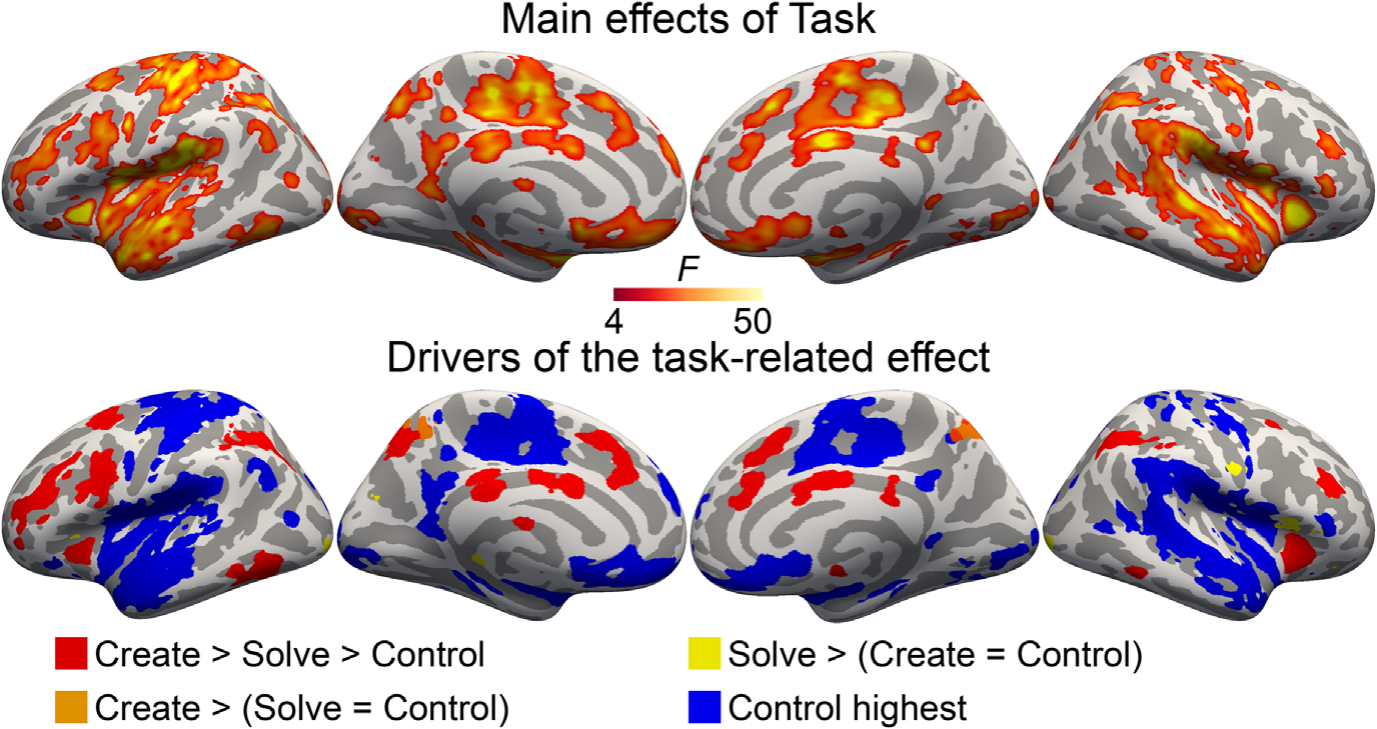
Main effects of task (initial cluster forming threshold P = 0.0001, permuted cluster significance P < 0.05, FWER‐corrected). In the bottom row, colours illustrate which direction of effects drove the significant ANOVA effects.

Main effects of Task were observed in multiple regions of the brain (Figure 3, top). The effect was due to a parametric task‐dependent modulation (create > solve > control; Figure 3 bottom, red) in the lateral prefrontal cortices, intraparietal sulcus (IPS), anterior insulae, inferior temporal gyrus (ITG), in the superior frontal sulci (SFS) and gyri (SFG), middle and anterior CG, precuneus. Note that while there were proportionally more complex calculations performed during the create task than during the solve task, very similar activations are observed when contrasting only simple calculations from both tasks (Figure S1 in Supplementary Materials). This suggests that the parametric effect is not merely due to calculation complexity but reflects a more general difference in task‐related processing (i.e., the cognitive operations involved in completing the tasks, including both domain‐general processes and processes of the ‘core’ systems involved in numerical problem‐solving).

The solve task was associated with the highest activity while no difference was observed between the create and control tasks in the right central sulcus and lateral fissure. The create task was associated with the highest activity while there was no significant difference between activity levels during the solve and control tasks in the left and right precunei.

As brain activity related to the control task is not in the centre of interest in the present study, we used a single colour to illustrate all regions where this task was associated with the highest activity, regardless of other possible activation differences between the tasks. These activations span wide regions in temporal, insular and opercular regions, in the pre‐ and post‐central gyri and central sulci bilaterally, as well as in medial regions including the CG, precuneus, subparietal sulcus, SFG, parahippocampal gyrus and medial prefrontal cortex.

**FIGURE 4:**
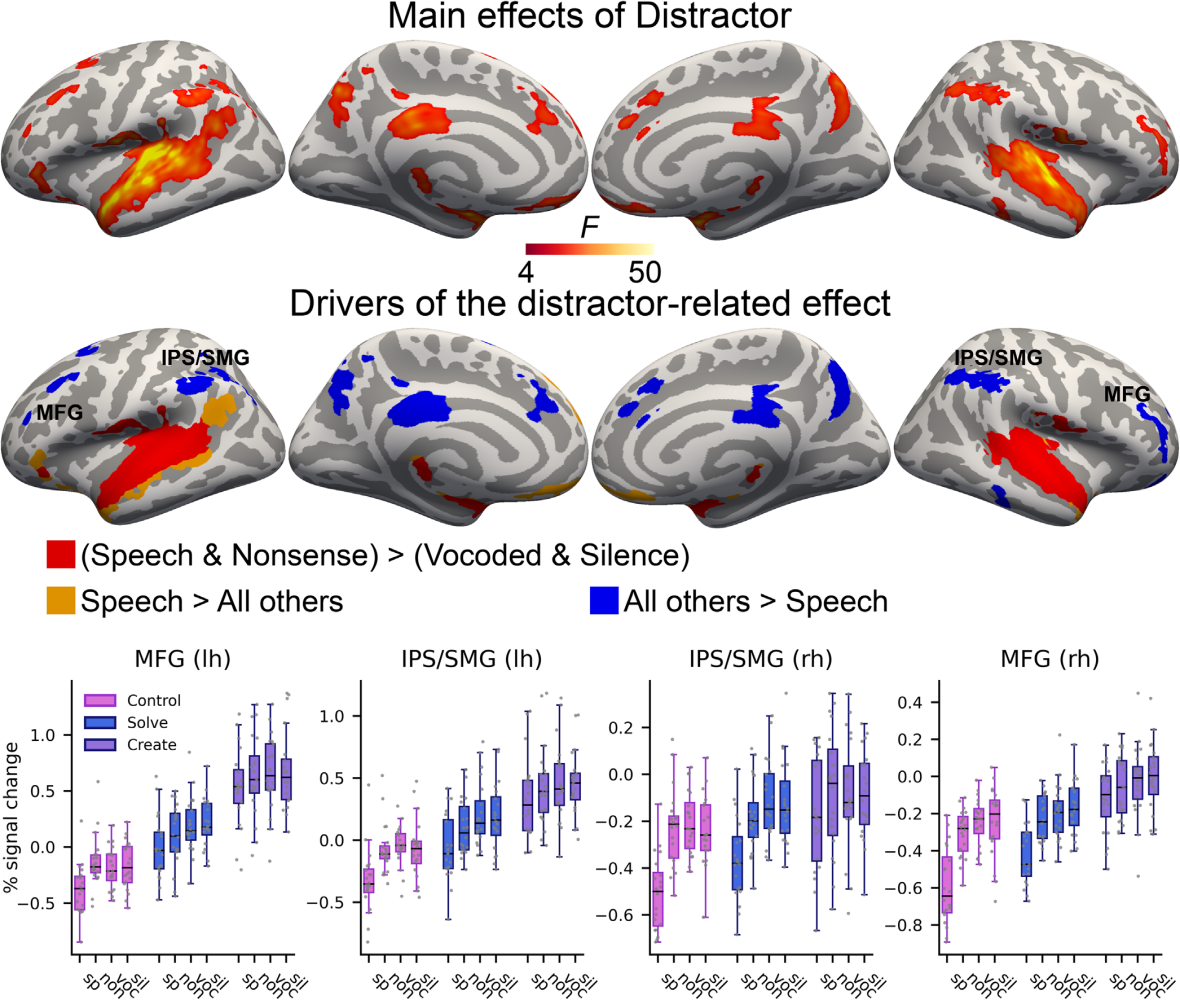
Main effects of distractor (top; initial cluster forming threshold P = 0.001, permuted cluster significance P < 0.05, FWER‐corrected). Middle: different colours illustrate which direction of effects drove the significant ANOVA findings. Bottom: to better understand the unexpected fronto‐parietal speech‐related deactivations, we extracted percent signal change values for each condition from these clusters (data points for individual subjects). Abbreviations: MFG, middle frontal gyrus; IPS, intraparietal sulcus; SMG, supramarginal gyrus; lh, left hemisphere; rh, right hemisphere; sp, speech; non, nonsense; voc, noise vocoded; sil, silence.

Main effects of Distractor were observed in temporal, posterior perisylvian, prefrontal, parietal and medial regions (Figure 4). In the superior temporal sulci and gyri (STG/STS) bilaterally and in the left IFG, the meaningful and nonsense speech distractors were both associated with higher activity than the other distractors. The meaningful speech distractor was uniquely associated with higher activity than all other distractors in the left STS and ventral AG, left IFG, ventromedial prefrontal cortex (vmPFC) and left SFG. The meaningful speech distractor was associated with less activity than all other distractors bilaterally in the middle frontal gyrus (MFG), supramarginal gyrus (SMG), dorsal AG and IPS, as well as in the right inferior temporal sulcus, and medially in the precuneus, parieto‐occipital sulcus, posterior precallosal sulcus, SFG and anterior and posterior CG. Because the fronto‐parietal deactivations during the meaningful speech were contrary to our hypothesis, we extracted and plotted percent signal change values from these clusters. These indicate that aside from meaningful speech being associated with the lowest activity, activity levels during the other distractor conditions did not markedly differ from each other in any task or cluster.

**FIGURE 5:**
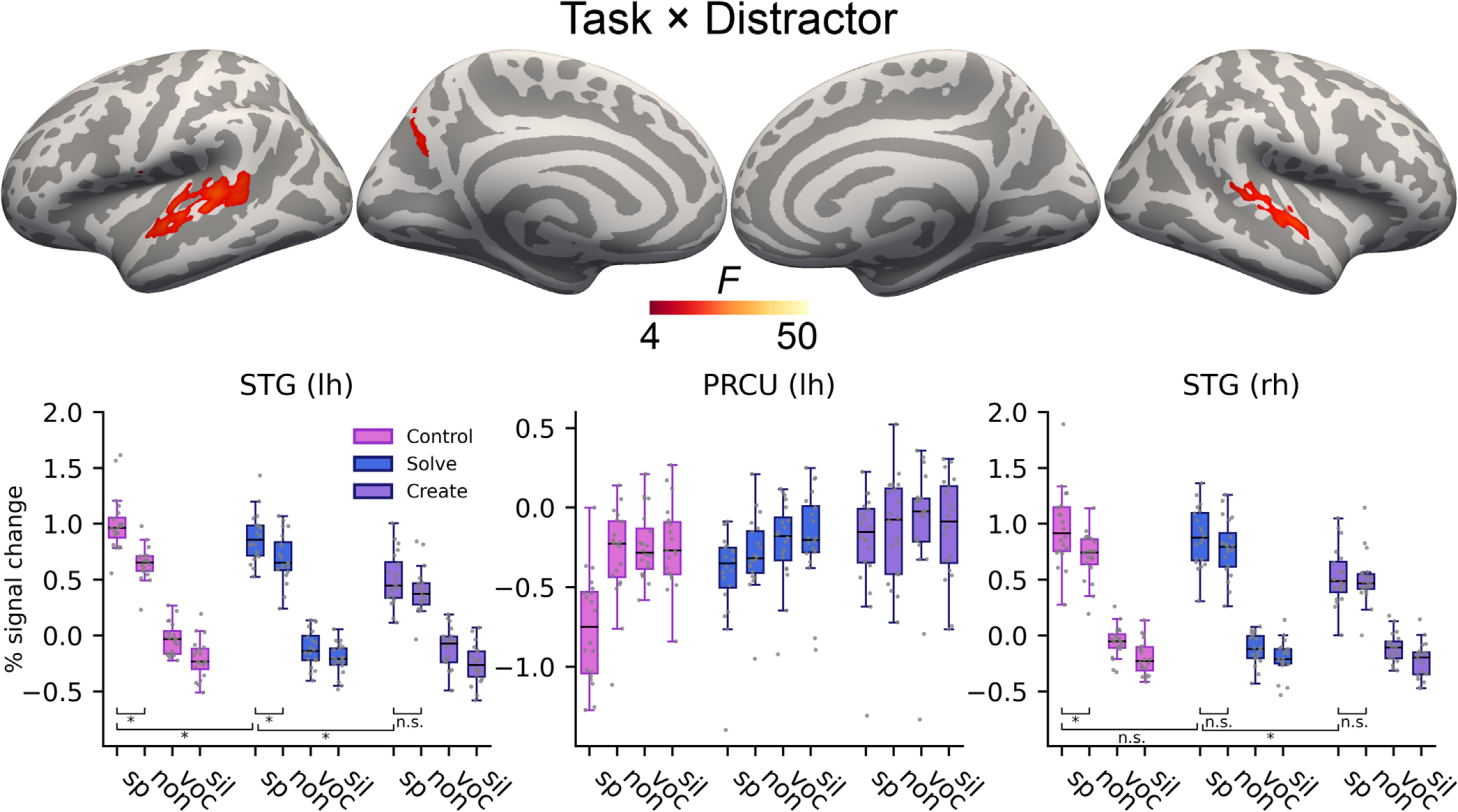
Significant interactions between task and distractor in the 3 × 4 whole‐brain ANOVA (top; initial cluster forming threshold P = 0.001, permuted cluster significance P < 0.05, FWER‐corrected). Plots show percent signal change values extracted from the clusters, with data points for individual subjects. Horizontal lines indicate the significance of the most relevant post‐hoc comparisons in the two STG clusters, that is, contrasts between the speech condition across each task pair, as well as differences between the speech and nonsense speech conditions within each task. The asterisks indicate significant differences (P < 0.05, Bonferroni corrected across all 15 comparisons in each cluster). Abbreviations: STG, superior temporal gyrus; PRCU, precuneus; lh, left hemisphere; rh, right hemisphere; sp, speech; non, nonsense; voc, noise‐vocoded; sil, silence.

Clusters with a significant interaction between Task and Distractor were observed in the auditory regions of the left STS/STG and in the right STG, as well as in the left precuneus/parieto‐occipital sulcus. The left and right plots in Figure 5 indicate that the AC interactions were primarily driven by the task factor modulating activity levels during the meaningful and nonsense speech conditions, but not during the noise‐vocoded and silent conditions. As indicated by the middle plot in Figure 5, the interaction in the left precuneus/parieto‐occipital sulcus was primarily driven by the meaningful speech distractor being associated with lowest activity during the control task, second lowest during the solve task and highest during the create task.

The interactions observed in the bilateral AC were further inspected with Bonferroni corrected post‐hoc *t*‐tests, because they pertained to the primary topic of this study. All *P*‐values reported below are Bonferroni adjusted by multiplying the original *P*‐value by 15, the number of tests within each cluster (if the resulting value exceeds 1, it is reduced to 1).

In the left AC, activity during the meaningful speech was higher during the control task than either of the other tasks (control vs. solve *t*_*19*_ = 3.63, *P* = 0.027, control vs. create *t*_*19*_ = 12.81, *P* < 0.001), and higher during the solve task than the create task (*t*_*19*_ = 10.70, *P* < 0.001). Activity during nonsense speech did not significantly differ between the control and solve tasks (*t*_*19*_ = −1.65, *P* = 1), but differed between the create task and the other tasks (control vs. create *t*_*19*_ = 8.65, *P* < 0.001, solve vs. create *t*_*19*_ = 10.80, *P* < 0.001). Activity during the noise‐vocoded distractor differed only between the control and solve tasks (*t*_*19*_ = 4.03, *P* = 0.011), but not between the control and create tasks (*t*_*19*_ = 2.68, *P* = 0.22) nor the solve and create tasks (*t*_*19*_ = −0.33, *P* = 1). During the silent distractor, there were no significant differences between any task pairs (control vs. solve *t*_*19*_ = 0.058, *P* = 1, control vs. create *t*_*19*_ = 1.79, *P* = 1, solve vs. create *t*_*19*_ = 1.31, *P* = 1). As for differences in activity levels between meaningful speech and nonsense speech within each task, these two conditions differed from each other during the control task (*t*_*19*_ = 7.85, *P* < 0.001) and the solve task (*t*_*19*_ = 4.46, *P* = 0.004), but not during the create task (*t*_*19*_ = 2.87, *P* = 0.15).

In the right AC, activity during meaningful speech was higher during the control task than the create task (*t*_*19*_ = 9.30, *P* < 0.001) and during the solve task than the create task (*t*_*19*_ = 10.74, *P* < 0.001), but did not differ between the control and solve tasks (*t*_*19*_ = 2.52, *P* = 0.311). The same pattern held true for activity during nonsense speech (control vs. solve *t*_*19*_ = −1.90, *P* = 1, control vs. create *t*_*19*_ = 8.35, *P* < 0.001, solve vs. create *t*_*19*_ = 8.25, *P* < 0.001). Activity during the noise‐vocoded distractor did not significantly differ between any task pair (control vs. solve *t*_*19*_ = 2.60, *P* = 0.262, control vs. create *t*_*19*_ = 1.85, *P* = 1, solve vs. create *t*_*19*_ = −0.43, *P* = 1), and the same held true for the silent distractor (control vs. solve *t*_*19*_ = 0.12, *P* = 1, control vs. create *t*_*19*_ = 0.85, *P* = 1, solve vs. create *t*_*19*_ = 0.60, *P* = 1). As for differences in activity levels during meaningful speech and nonsense speech within each task, these two conditions differed from each other during the control task (*t*_*19*_ = 4.94, *P* = 0.0014), but not during the other tasks (solve *t*_*19*_ = 2.14, *P* = 0.68, create *t*_*19*_ = 0.78, *P* = 1).

**FIGURE 6:**
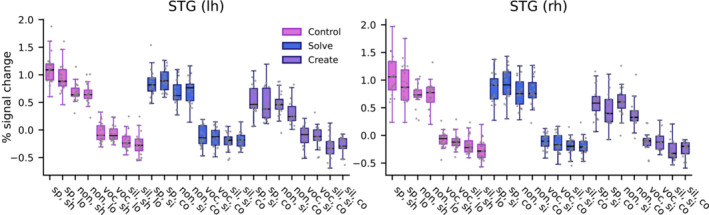
Percent signal change values extracted from the two auditory cortex clusters with significant interactions between task and distractor, further partitioned between simple and complex trials (short and long trials in the control task; data points for individual subjects). Linear mixed models indicated a significant interaction between task, distractor, and complexity in both clusters. Abbreviations: STG, superior temporal gyrus; lh, left hemisphere; rh, right hemisphere; sp, speech; non, nonsense; voc, noise‐vocoded; sil, silence; sh, short; lo, long; si, simple; co, complex.

To further examine whether increasing calculation complexity led to attenuated AC activity within the tasks, percent signal change values from these ROIs were also extracted separately for each combination of task, distractor and calculation complexity (from the second GLM described in section 2.8.1; Figure 6). LMMs conducted on these values indicated that in both clusters, there was a significant main effect of Calculation complexity (left AC: *F*_1,25.4_ = 16.08, *P* < 0.001: right AC: *F*_1,21.3_ = 15.14, *P* < 0.001), but also significant interactions between Task, Distractor and Calculation complexity (left AC: *F*_6,221.4_ = 13.58, *P* < 0.001; right AC: *F*_6,236.7_ = 18.85, *P* < 0.001). As indicated by the plots in Figure 6, while AC activity during the speech distractors appears at least slightly attenuated in complex vs. simple expressions/calculations in the control and create tasks, the same does not hold for the solve task.

#### 3.2.2 Exploratory analysis of creative vs. routine arithmetic performance: effects of calculation complexity

**FIGURE 7:**
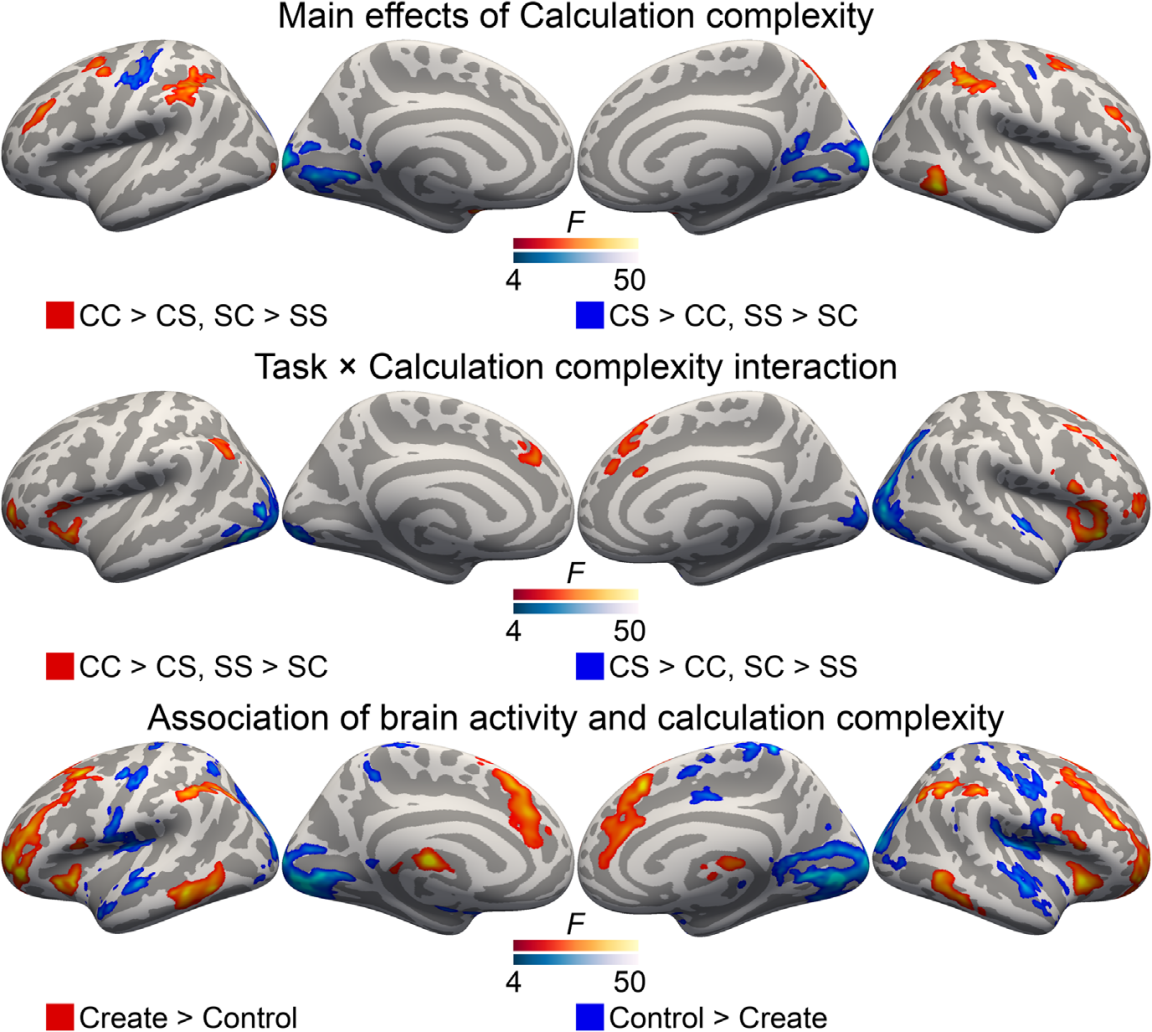
Exploratory analyses of differences between ‘routine’ and ‘creative’ arithmetic calculation. Top and middle: Main effects of calculation complexity and interactions between task and calculation complexity from the 2 × 2 ANOVA with factors task (solve, create) and calculation complexity (simple, complex). Please refer to figures S2 and S3 in the supplementary materials for detailed plots of activity in the different experimental conditions in all clusters. Bottom: clusters with significant associations between brain activity and calculation complexity in the create task (red) and expression length in the control task (blue). For all images: initial cluster forming threshold P = 0.001, permuted cluster significance P < 0.05, FWER‐corrected. Abbreviations: CC, create complex; CS, create simple; SC, solve complex; SS, solve simple.

Main effects of calculation complexity were observed in multiple brain regions (Figure 7, top). Complex calculations were associated with higher activity than simple calculations in the MFG, SMG and IPS bilaterally, as well as in the left precentral gyrus, right posterior ITG and right SFS (Figure 7, top, red). Conversely, simple calculations were associated with higher activity in occipital regions, bilateral central sulcus and the left and right putamen (Figure 7, top, blue).

Interactions between task and calculation complexity were also observed in widespread regions of the brain (Figure 7, middle). In bilateral anterior MFG, insula, left AG, right posterior MFG and medial frontal regions, the interaction was due to higher activation during complex than simple calculations in the create task, with the reverse effect or no effect in the solve task. In the occipital cortices and right STG, the interaction was due to higher activation during complex than simple calculations in the solve task, and vice versa in the create task. Please refer to Figures S2 and S3 in the Supplementary Materials for detailed plots from each cluster, as well as Figure S4 for an illustration of subcortical activations that are poorly or not at all visible in the surface renderings.

Complex calculations in the solve task included two arithmetic operators, but the participants were free to create even more complex calculations in the create task (i.e., with three or more operators). Because of the confounding effect this might introduce, the interaction analysis described above did not include these most complex create task calculations. However, these calculations were included in the analysis on associations between calculation complexity and brain activity, which aimed to identify brain regions where activity scaled with increasing calculation complexity (please refer to section 2.8.1 for details). To control for effects related merely to inputting longer expressions, activity during the control task was similarly modelled as a function of expression length, and these were then contrasted. This analysis indicated that complex calculations in the create task were associated with increased brain activity in the bilateral prefrontal regions, insulae, SMG, IPS, AG, anterior CG, SFG and inferior posterior temporal regions, as well as subcortically in the thalamus. Conversely, longer expressions in the control task were more highly associated with activity in the bilateral occipital regions, central sulci, temporal regions, middle and posterior medial regions.

## 4 DISCUSSION

### 4.1 The tasks recruited a fronto‐parieto‐temporal network of brain regions parametrically

A network of fronto‐parieto‐temporal brain regions including the MFG, anterior CG, anterior insula, IPS and left posterior ITG was parametrically activated by the present experimental tasks (Figure 3): highest activity was observed during the creative arithmetic task, the second highest during the routine calculation task and lowest activity during the control task. This result remained also when contrasting only simple calculations from the two arithmetic tasks (Figure S1), indicating that the activity differences between the creative and routine calculation tasks are not explained only by there being proportionally more complex answers in the creative task (Figure 2B). These results are in line with previous research demonstrating higher activation in these networks in more demanding task conditions, both in cognitive tasks in general and in arithmetic tasks specifically (Arsalidou & Taylor, 2011; Dehaene et al., 2004; Fedorenko et al., 2013; Grotheer et al., 2018). Thus, these effects are not informative on any single task‐related cognitive processes. Importantly, however, they are consistent with our a priori assumptions that the tasks differ in difficulty; that the creative task is more demanding than the routine calculation task (largely because of the additional demands it poses on various aspects of cognitive control, including working memory and cognitive flexibility); and that the control task is less demanding than either of the other tasks. Furthermore, these results indicate that although the present experimental design contained certain distinctive features (i.e., participants used a trackball to input answers in a self‐paced manner), the task‐related neural effects are consistent with what was anticipated. This supports the validity of the present tasks in studying the effects of increasing cognitive demands on task‐irrelevant auditory processing.

### 4.2 Task‐irrelevant speech processing was disproportionately attenuated by increasing task demands

The auditory regions of the STS and STG were most activated by the two speech distractors, and the meaningful speech distractor was further associated with the highest activity in regions including the left IFG, left posterior STS and ventral AG (Figure 4). This was expected, as these regions are associated with aspects of speech and semantic processing (Binder et al., 2009; Hickok & Poeppel, 2007). They are also consistent with earlier research showing that speech and its meaning can be processed to an extent also in the absence of attention (Beaman et al., 2007; Har‐shai Yahav & Zion Golumbic, 2021; Rämä et al., 2012; Röer et al., 2017b; Ylinen et al., 2022).

Importantly, however, interactions between task and distractor revealed that activity in the bilateral AC was differentially modulated depending on task across the four distractor conditions: while activity during the noise‐vocoded and silent distractors differed only slightly or not at all between the different tasks, activity related to the two speech distractors showed a marked attenuation as the cognitive demands of the tasks increased (Figure 5). These results corroborate earlier findings demonstrating that the processing of task‐irrelevant auditory information can be suppressed as the cognitive demands of a primary task increase (Berti & Schröger, 2003; Brockhoff et al., 2022; Marsh et al., 2015; Parmentier, 2014; Sörqvist et al., 2016). Moreover, the attenuation effect was more pronounced for meaningful than for nonsense speech, especially in the left AC. Specifically, post‐hoc tests revealed that in the left AC, activity in the meaningful speech conditions decreased from the control task to the solve task to the create task, and while activity during meaningful speech differed significantly from activity during a nonsense speech in the control and solve tasks, this difference was no longer significant in the create task. These results extend previous findings by demonstrating that although meaningful speech, as a complex and ecologically relevant stimulus, elicited the highest levels of activity in conditions with lower cognitive demands, its processing could also be attenuated to the greatest extent as the task demands increased.

The present results on the attenuation of task‐irrelevant processing are broadly consistent with *Load Theory* (Lavie, 2005; Lavie & Tsal, 1994), which holds that task‐irrelevant information can only be processed when sufficient attentional resources are available. It is nonetheless interesting to note that, in our study, activity related to meaningful speech was attenuated more than activity related to nonsense speech, which was a physically very similar stimulus but contained no meaningful words. This difference suggests that higher‐level processes, such as semantic processing, may be particularly susceptible to suppression when cognitive demands are high. Moreover, while previous research indicates that meaningful speech can disrupt performance in cognitive tasks more than meaningless speech (Hughes & Marsh, 2020; LeCompte et al., 1997; Oswald et al., 2000; Röer et al., 2019; Vasilev et al., 2018), we did not observe significant behavioural effects of distractor type in the present study. This aligns with earlier findings suggesting that the disruptive effect of speech may depend on factors such as its emotional salience and its relevance to the task at hand (Marsh et al., 2009, 2014; Röer et al., 2017a). Thus, it may be that the fact that the present speech stimuli were neither emotionally salient nor directly related to the tasks made it relatively easy to attenuate the processing of their semantic content to a level that did not impair task performance–an interpretation that is consistent with the observed AC attenuations.

To explore whether increasing calculation complexity (i.e., calculations with one vs. two arithmetic operators) also led to attenuated AC activity, we performed additional ROI analysis partitioning events by calculation complexity as well as by task and distractor type. As indicated by the plots in Figure 6, increasing calculation complexity in the create task appeared to induce attenuated AC activity during the speech distractors, although to a lesser extent during meaningful than nonsense speech. During the control task, AC attenuation was evident during meaningful speech. These results are consistent with increasing task demands leading to attenuated processing of task‐irrelevant auditory information. However, linear mixed models on ROI data from both hemispheres also indicated an interaction between task, distractor and calculation complexity. This was due to the somewhat surprising observation that during the solve task, increasing calculation complexity did not induce AC attenuations (Figure 6). It could be that even complex calculations in this task were not particularly difficult for our university student participants, but the result nonetheless suggests that task difficulty may not be the sole factor driving the present AC attenuations.

One possible factor underlying the AC attenuations could be increased motor activity, resulting simply from inputting longer expressions (Aliu et al., 2009; Schneider et al., 2014; Wikman et al., 2015). This effect would be more significant during the control and create tasks, because solutions to simple and complex solve task calculations did not necessarily differ in length (e.g., simple: 4+3 = 7, complex: 2×3–3 = 7), but did so by design in short vs. long expressions in the control task and simple vs. complex calculations in the create task. However, this explanation does not account for the differences observed between tasks, nor for differences between distractor conditions within tasks. Another factor potentially explaining some of these results is task novelty, as the solve task was arguably more familiar to the participants than the other tasks. Novel tasks in general require higher levels of metacognitive processing, which relies on partially distinct neural networks from other types of cognitive control (Chein & Schneider, 2012; Fleming & Dolan, 2012; Morales et al., 2018). Thus, metacognitive processing might induce differential AC attenuations as well. However, this explanation also falls short of fully explaining the present pattern of results, as it does not account for diminished activity during the solve task compared to the more novel control task. It seems likely that all these factors may play roles in the observed AC attenuations. Importantly, however, the absence of an attenuation effect between simple and complex calculations in the solve task does not invalidate the primary conclusion that task‐irrelevant auditory processing can be more strongly attenuated for speech than for meaningless but physically well‐matched stimuli.

### 4.3 Fronto‐parietal regions displayed deactivation related to meaningful speech

In several previous studies, attenuated AC activity related to the processing of task‐irrelevant stimuli has been observed together with increased activation in frontal and/or parietal regions (Causse et al., 2022; Ghatan et al., 1998; Gisselgård et al., 2004; Kulasingham et al., 2021; Sörqvist et al., 2016). Unexpectedly, however, the present study showed deactivation in fronto‐parietal regions under conditions with meaningful speech compared to other sounds (Figure 4). These deactivations partially overlap with the task and complexity‐related effects (Figures 3 and 7), and the plots in Figure 4 further indicate a parametric task‐related activity modulation in these clusters. This shows that speech‐related deactivations occur in regions where task‐relevant processing also takes place. What might cause this discrepancy between the earlier and present results?

A possible explanation relates to the type of the distracting stimulus. Most previous studies have used relatively simple distractor stimuli (e.g., tones or single words), whereas the present study used continuous meaningful speech, a highly complex and ecologically relevant stimulus type. This suggestion is supported by the fact that at least one earlier study has observed similar deactivations in the left dlPFC and inferior parietal regions when contrasting working memory performance under task‐irrelevant continuous meaningful speech vs. aircraft noises (Sætrevik & Sörqvist, 2015). Given the overlap between the deactivations and task‐related activations, one obvious suggestion would be that these deactivations reflect disrupted task‐related processing. However, in the absence of behavioural effects of distractor type (in the present study as well as in the study by Sætrevik and Sörqvist), this interpretation is not straightforward. Another suggestion that has been put forth to explain similar effects is that the deactivations might reflect a reduced need for top‐down control for task processing, stemming from increased arousal (Smucny et al., 2013). In any case, it should be noted that whether the deactivations reflect one of these explanations or some other underlying factor, the results nonetheless suggest that task‐irrelevant meaningful speech leads to markedly different effects than simpler distractors in the brain's control networks. This highlights the importance of ecologically relevant stimuli in future studies on auditory distraction.

Speech‐related deactivations were also observed in the posterior CG and pericallosal sulcus, as well as in the left precuneus (Figure 4). In the left precuneus, an interaction was also observed, as the meaningful speech was associated with the least activity during the control task (Figure 5, middle). These are likely accounted for by the fact that these regions make part of the default mode network (DMN), where deactivations are often observed in more challenging experimental conditions, and where specifically speech‐related deactivations have also been observed (Seghier & Price, 2012).

### 4.4 Exploration of brain activity in creative vs. routine arithmetic performance

As discussed above, much of the activity differences between creative and routine arithmetic performance were due to a parametric task‐dependent modulation observed in regions where ‘task‐positive’ activity is typically observed (Figure 3). Concordantly, the main effects of calculation complexity were also observed in largely overlapping portions of the bilateral MFG and IPL (Figure 7, top). Thus, these effects may be mostly accounted for by increasing cognitive demands in general and are not readily interpretable as anything specific to either of the arithmetic tasks.

To identify effects more specifically associated with creative arithmetic problem‐solving, we examined interactions between task and calculation complexity, as these can reveal activity independent of mere task difficulty. In the creative arithmetic task, participants could create equations more complex than any included in the routine calculation task (i.e., containing more than three operands). To avoid the potential confounds this might introduce, we excluded the most complex creative task calculations from the primary interaction analysis. However, we included them in a separate analysis that aimed to identify positive associations between brain activity and calculation complexity.

Interactions due to increasing activity in complex vs. simple calculations in the creative task and decreasing activity for complex vs. simple calculations in the routine calculation task were observed in multiple regions including the anterior prefrontal regions, anterior insulae and anterior CG (Figure 7, middle, Figure S3). These regions were strongly implicated also by the analysis on associations between brain activity and calculation complexity, which included also the most complex calculations (Figure 7, bottom). Previous research has shown the anterior MFG to be involved in conditions where abstract knowledge of task goals and maintaining a superordinate goal while completing a sub‐goal are necessary (Badre & Nee, 2018; Koechlin et al., 1999; Nee & D'Esposito, 2016; Nitschke et al., 2017; Szczepanski & Knight, 2014). Individual differences in these regions have also been shown to be associated with differences in working memory control (Minamoto et al., 2015). The insula, inferior IFG and anterior CG are also involved in cognitive control, goal‐directed task performance and metacognitive monitoring (Bellon et al., 2020; Dosenbach et al., 2006; Jiang et al., 2015; Wu et al., 2019), as well as in arithmetic processing in specific (Arsalidou & Taylor, 2011). These functions plausibly relate to the interactions observed here, as creating complex equations requires maintaining the goal of reaching a target answer while pursuing different strategies to get there.

A similar interaction to the ones discussed above was observed in the left AG (Figure 7, middle; Figure S3), which was also implicated by the analysis on associations between calculation complexity and brain activity. The role of the left AG in arithmetic processing has received considerable interest over the years (for a review, see Sokolowski, Matejko, & Ansari, 2023). Previous research indicates that arithmetic problems that are solved using fact retrieval activate the left AG more than complex, procedurally solved problems (Sokolowski, Hawes, & Ansari, 2023). Since the AG makes part of the DMN, interpreting these results is not straightforward, because DMN regions often deactivate in more demanding conditions (Humphreys et al., 2015). Yet, the AG has also been suggested to be more activated during ‘higher’ mathematical problem‐solving than during routine calculation (Cheng et al., 2022; Liu et al., 2019; Zhou et al., 2018). The present results seem to concur with both of these notions, as the interaction was due to lower activity during complex than simple routine calculations, but higher activity during complex than simple calculations in the creative arithmetic task. Given the proposition that the creative arithmetic task requires more profound utilization of arithmetic knowledge especially when creating complex equations (McMullen et al., 2016), the present AG results could in part reflect its role in supporting the retrieval and integration of arithmetical knowledge for complex problem‐solving. Clearly, however, this suggestion remains speculative and warrants further research.

Taken together, the present results indicate that the creative task is associated with distinct activations in brain regions implicated in multiple aspects of cognitive control, and might also recruit the AG in the retrieval and manipulation of arithmetic knowledge. Future studies could benefit from using this task in examining these in the context of arithmetic problem‐solving, and also in targeting individual differences in these neurocognitive functions.

### 4.5 Limitations and future directions

Naturalistic speech is a highly dynamic stimulus, and an important limitation of the present study is that fMRI lacks the temporal resolution to track its processing precisely. Furthermore, it should be acknowledged that the present auditory stimuli were presented amid continuous scanner noise, which may have affected the results (e.g., meaningful speech might have been easier to ignore than it would be when presented as the sole auditory stimulus). Thus, methods with higher temporal resolution and no accompanying noise (e.g., electro‐ and magnetoencephalography) could be used to provide more insight on the details of how task‐irrelevant speech processing is modulated depending on task demands and features of the distractor itself. Future studies could also focus on situations where the attenuation mechanism fails, potentially resulting in disrupted behavioural performance and greater distractibility. This would help clarify the boundary conditions under which cognitive control of distraction succeeds or breaks down.

Secondly, the sample size of the present study was determined to be appropriate only for examining within‐subjects effects relating to task‐irrelevant auditory processing and numerical tasks. It is likely, however, that important individual differences exist in these functions, and future studies with larger sample sizes are needed to uncover these effects. For example, studies could examine associations between behavioural performance, attenuation effects in the auditory regions and activation patterns in the fronto‐parietal control network, to uncover how individual differences are reflected in the interactions between cognitive load and auditory distraction. Moreover, the findings of this study may also have implications for clinical populations that exhibit atypical auditory or distractor‐related processing. For instance, individuals with autism spectrum disorder often display altered auditory processing, particularly with complex and socially relevant stimuli like speech (O'Connor, 2012). Similarly, those with attention deficit hyperactivity disorder tend to exhibit greater distractibility, which is also reflected in the underlying neural responses (Gumenyuk et al., 2005; Oja et al., 2016). The present findings demonstrate differential processing of task‐irrelevant meaningful speech, compared to meaningless stimuli, in both the auditory system and fronto‐parietal control networks, suggesting that the ecological relevance of stimuli should be carefully considered also in future clinical studies. This might also be relevant for studies on hidden hearing loss, where individuals often have difficulty processing sounds in noisy environments despite normal hearing thresholds (Plack et al., 2014).

Finally, while the present arithmetic tasks are realistic in that they involve numerical processing together with multiple cognitive control processes, this comes with the compromise that task contrasts cannot reliably reveal any single task‐related process. Moreover, while the categorization of simple and complex calculations in the present study closely follows earlier ones (e.g., Sokolowski, Hawes, & Ansari, 2023), there are also differences in, for example, how different arithmetic operations are processed in the brain (Arsalidou & Taylor, 2011). This results in some heterogeneity in the present ‘simple’ and ‘complex' calculations. This heterogeneity does not represent a fatal flaw, given the consistencies between our exploratory analyses with earlier findings (e.g., effects in the left AG). Yet, it is important to note that further hypothesis‐driven studies are needed to confirm and more deeply understand the effects related to the creative arithmetic task.

## 5 CONCLUSIONS

We examined the neural effects associated with the processing of task‐irrelevant auditory stimuli during cognitive tasks varying in their demands. Our results add to previous findings by indicating that although task‐irrelevant meaningful speech was associated with the highest levels of activity in conditions with low cognitive demands, its processing could also be attenuated to the greatest extent as task demands increased. Contrary to previous results, however, meaningful speech was also associated with deactivation in fronto‐parietal regions. Thus, ecologically relevant stimuli, when used as task‐irrelevant distractors, appear to have distinct impacts on the brain's control networks. This should be considered in future studies on the cognitive control of auditory distraction. Finally, we explored differences in the brain activations underlying routine vs. creative arithmetic performance. While initial, our results suggest that the creative arithmetic task could prove useful in studying the neural basis of mathematical problem‐solving and individual differences therein.

## AUTHOR CONTRIBUTIONS

AY: Conceptualization, investigation, data curation, formal analysis, visualization, writing – original draft, writing – review & editing. MHS: Conceptualization, supervision, writing – review & editing, funding acquisition. JM: Conceptualization, writing – review & editing. EL: Conceptualization, writing – review & editing. PW: Conceptualization, formal analysis, writing – review & editing. KA: Conceptualization, supervision, writing – review & editing, funding acquisition.

## CONFLICT OF INTEREST STATEMENT

The authors have no conflicts of interest to disclose.

### PEER REVIEW

The peer review history for this article is available at https://www.webofscience.com/api/gateway/wos/peer-review/10.1111/ejn.16616.

## ETHICAL STATEMENT

All participants gave written informed consent and were monetarily compensated for their participation (15 €/h). The experiment was conducted in accordance with the Declaration of Helsinki and the experimental protocol was approved by the Ethics Committee of the Hospital District of Helsinki and Uusimaa, Finland.

## Supporting information


**Table S1.** Sets of calculations used in the solve task.
**Table S2.** Sets of target answers and given numbers used in the create task.
**Table S3.** Sets of numerical expressions used in the control task. Table S4. Additional information on behavioural performance: observed frequencies, durations and proportions of correct solutions by trial type. The frequencies are given per experimental run (summed over the different distractor conditions), because in GLMs 3 and 4 (described in section 2.8.1), the trials were modelled across all distractor conditions, as there were no significant effects of distractor type on behavioural performance.
**Figure S1.** Contrast between simple calculations in the create and solve tasks. Note that the regions activated more highly by the simple create calculations largely overlap with the regions where a parametric task‐dependent modulation was observed in the analysis of the main effects of the task (Figure 3, main text).
**Figure S2.** Main effects of Calculation complexity (initial cluster forming threshold P = 0.001, permuted cluster significance P < 0.05, FWER‐corrected) from the 2 × 2 ANOVA with factors task (solve, create) and calculation complexity (simple, complex). Y‐axis depicts percent signal change. Abbreviations: MFG, middle frontal gyrus; SFS, superior frontal sulcus; PreCG, precentral gyrus; CS, central sulcus; IPL, inferior parietal lobule; OccP, Occipital pole; Occ, occipital; pIPS, posterior inferior parietal sulcus; pITG, posterior inferior temporal gyrus; PUT, putamen; lh, left hemisphere; rh, right hemisphere; s, simple, c, complex (in the control task, these refer to short and long expressions, respectively).
**Figure S3.** Interactions between Task and Calculation complexity (initial cluster forming threshold P = 0.001, permuted cluster significance P < 0.05, FWER‐corrected) from the 2 × 2 ANOVA with factors Task (solve, create) and calculation complexity (simple, complex). Abbreviations: aMFG, anterior middle frontal gyrus; INS, insula; AG, angular gyrus; lat Occ, lateral occipital; Occ, occipital; SFG, superior frontal gyrus; ACC, anterior cingulate cortex; STG, superior temporal gyrus; pMFG, posterior middle frontal gyrus; IFG, inferior frontal gyrus; TP, temporal pole; lh, left hemisphere; rh, right hemisphere; s, simple, c, complex (in the control task, these refer to short and long expressions, respectively).
**Figure S4.** Left: a coronal slice from the analysis on the main effects of Calculation complexity displaying clusters in the left and right putamen. Right: a coronal slice from the analysis on associations of brain activity and calculation complexity displaying a cluster in the thalamus.
**Table S5.** Clusters observed in the analysis on the main effects of Task in the 3 × 4 ANOVA with factors Task (solve, create, control) and Distractor (speech, nonsense, vocoded, silence). Coordinates are given in the MNI space.
**Table S6.** Clusters observed in the analysis on the main effects of Distractor in the 3 × 4 ANOVA with factors Task (solve, create, control) and Distractor (speech, nonsense, vocoded, silence). Coordinates are given in the MNI space.
**Table S7.** Clusters observed in the analysis on the interaction between Task and Distractor in the 3 × 4 ANOVA with factors Task (solve, create, control) and Distractor (speech, nonsense, vocoded, silence). Coordinates are given in MNI space. Table S8. Clusters observed in the analysis on the main effects of Calculation complexity in the 2 × 2 ANOVA with factors Task (solve, create) and Calculation complexity (simple, complex). Coordinates are given in MNI space.
**Table S9.** Clusters observed in the analysis on the interaction between Task and Calculation complexity in the 2 × 2 ANOVA with factors Task (solve, create) and Calculation complexity (simple, complex). Coordinates are given in MNI space.
**Table S10.** Clusters observed in the analysis on the associations between brain activity and calculation complexity. Coordinates are given in MNI space.

## Data Availability

Group‐level fMRI, region of interest and behavioural data that support the findings of this study are available in the Open Science Framework, https://osf.io/v6zp5/. The conditions of our ethics approval do not permit public archiving of participant‐level fMRI data. Readers seeking access to these data should contact the lead author (AY).
