# Supplementary material for: Attenuated processing of task‐irrelevant speech and other auditory stimuli: fMRI evidence from arithmetic tasks
Source: Eur J Neurosci. 2024 Nov 25;60(12):7124–47. doi: 10.1111/ejn.16616 (PMC11647424; doi:10.1111/ejn.16616)
Supplement: Supplementary file 1 — Table S1. Sets of calculations used in the solve task. Table S2. Sets of target answers and given numbers used in the create task. Table S3. Sets of numerical expressions used in the control task. Table S4. Additional information on behavioural performance: observed frequencies, durations and proportions of correct solutions by trial type. The frequencies are given per experimental run (summed over the different distractor conditions), because in GLMs 3 and 4 (described in section 2.8.1), the trials were modelled across all distractor conditions, as there were no significant effects of distractor type on behavioural performance. Figure S1. Contrast between simple calculations in the create and solve tasks. Note that the regions activated more highly by the simple create calculations largely overlap with the regions where a parametric task‐dependent modulation was observed in the analysis of the main effects of the task (Figure 3, main text). Figure S2. Main effects of Calculation complexity (initial cluster forming threshold P = 0.001, permuted cluster significance P < 0.05, FWER‐corrected) from the 2 × 2 ANOVA with factors task (solve, create) and calculation complexity (simple, complex). Y‐axis depicts percent signal change. Abbreviations: MFG, middle frontal gyrus; SFS, superior frontal sulcus; PreCG, precentral gyrus; CS, central sulcus; IPL, inferior parietal lobule; OccP, Occipital pole; Occ, occipital; pIPS, posterior inferior parietal sulcus; pITG, posterior inferior temporal gyrus; PUT, putamen; lh, left hemisphere; rh, right hemisphere; s, simple, c, complex (in the control task, these refer to short and long expressions, respectively). Figure S3. Interactions between Task and Calculation complexity (initial cluster forming threshold P = 0.001, permuted cluster significance P < 0.05, FWER‐corrected) from the 2 × 2 ANOVA with factors Task (solve, create) and calculation complexity (simple, complex). Abbreviations: aMFG, anterior middle frontal gyrus; INS, i [file EJN-60-7124-s001.pdf]

Supplementary materials

This document contains supplementary materials for the main manuscript.

Included here are:

- Sets of all calculations used in the solve task, all target answers and given numbers used in the create task, and all expressions used in the control task (Tables S1-S3)

- The observed frequencies, durations, and proportions of correct solutions by trial type (Table S4)

- Result images supporting the claims made in the main manuscript but not included there (Figures S1-S4)

- Result tables from all fMRI analyses (Tables S5-S10)

- A detailed description of the (f)MRI data preprocessing steps, as provided by *fMRIPrep*.

Supplementary data and codes can be found on the Open Science Framework (https://osf.io/v6zp5/).

Table S1. Sets of calculations used in the solve task.

Set																				
**1**	4+3	7-7	2·5-3	48:6	4·4	6:(5-2)	48:8	3+3	8+10	8·2	(2+6):4	6+1·2	3+2	5·9	1-1	36:6	5-1	6·2	56:7	7-1
**2**	7+4	40:10	4-3	8-3+4	5·(1+2)	9·9	8-6	5+1·3	8·8	30:3	2+3	30:5	3+5	10:5	19-9	3-2	9:(5-2)	3·8	6+2	5·1
**3**	18:2	3·(4+2)	7-3	1+1	7+2·3	4·7	3·9	6·4	4·(3+1)	10-10	5-2+4	1+2	9-3	10-4	1·1	42:7	40:4	4+1	45:9	6+6
**4**	1+9	10:2	5+2-4	4-1	4:(6-2)	7·8	35:7	9+5	3·7	24:3	8-7	35:5	7·4	10-2	9+6	6:(3-1)	7·1	5+10	9-1	6-1+3
**5**	18:3	4·6	5+2·2	20-10	8:(5-1)	1+4	8+8	3·5	7+7	20:4	9·6	2·5	6-4-1	(5-2)·3	6-3	2-1	18:9	11-10	25:5	2+8
**6**	9-5	4+6	4+2-3	(5+1):2	1·2	49:7	4·(3+2)	12:3	54:9	4+5	7·5	2·9	6·7	24:4	6-1	5+4-2	5-2	6+8	6-6	7+1
**7**	2+1	5·6	4-2	3+6:3	63:7	(4-1)·2	6·8	9-5+2	64:8	(2+4):2	6-4	7+2	9+8	8-3	2·6	28:4	24:8	9-6	2+2	5·4
**8**	3·(2+1)	7+1-3	6:3	8·7	2+4	18-8	9-8	(3-2)·4	3+6	21:7	12:2	10·3	20:2	6·1	10-9	3+2+2	5+2	7-5	10+10	5·7
**9**	5+6	21:3	7-4+2	2·4	8-5	(4+2):2	8·3	6+1-4	10-8	16:2	6+4	4:(3-1)	9+3	14-7	7·7	6:2	1+6	3·2	40:5	9-9
**10**	(3-1)·4	8:2	4+3-1	8+4	1·9	6-2	(4-2)·3	3-3	8-3+1	5·3	5+4	6+5	45:5	7·6	9-7	8·1	7-2	8+7	15:3	4:2
**11**	7+3-1	3·4	3-1	(3+1):4	20:10	6+1	8:2+4	50:10	(5-1)·2	3+4	4·2	7-6	13-3	4·3	1+5	8:4	4+4	12-2	27:3	3·3
**12**	9-4	6+3-2	4·9	3·(1+2)	2+9	12:6	9+4	7-4	24:6	6·5	14:2	(5+3):2	8-4	9·8	54:6	5+5	10-6	9:3-1	7·2	9+1
**13**	5-4	5+3-2	15:5	2+6	(4-1)·3	5·2	8-2	32:4	36:9	8:(3-1)	17-7	11-1	1+3	1·4	6+7	2·2	2·5+1	12:4	1+7	5·5
**14**	7·9	8-8	(2+2):2	5+1	5+4:2	30:6	2·(1+3)	3·6	18:6	6+3	27:9	7-2+4	7+5	8-1	1+8	10-5	7·3	32:8	2·8	10-1
**15**	16-6	3+8	3·(4+1)	5-4+2	9:3	8·6	20:5	2-2	10·1	6-5	4+2	3+1	3+7	8·5	8-1+3	7-(2·3)	40:8	28:7	6·6	15-5
**16**	(2-1)·4	6-3+1	36:4	10-7	7+9	3·1	2·3	9+9	4-4	8+5	16:8	8:(6-2)	10-3	4·8	30:10	6·3	8:4+2	5-5	50:5	8+2

Table S2. Sets of target answers and given numbers used in the create task.

**Set**	**Target**	**Given 1**	**Given 2**	**Given 3**	**Given 4**	**Given 5**
**1**	8	2	4	6	16	24
**2**	15	3	5	10	20	30
**3**	16	2	4	8	12	32
**4**	20	2	4	5	15	40
**5**	12	2	4	6	16	24
**6**	6	2	4	12	16	24
**7**	4	2	8	10	16	20
**8**	30	2	3	6	10	15
**9**	3	2	6	9	12	18
**10**	5	2	3	10	15	25
**11**	18	1	2	3	5	6
**12**	24	2	3	4	6	8
**13**	10	2	5	15	20	25
**14**	21	1	2	3	7	14
**15**	25	2	5	10	15	50
**16**	9	2	3	5	6	18

Table S3. Sets of numerical expressions used in the control task.

Set																				
1	1	(48)7	29	08627	068	8	2	653	15	0	881	36	7	(65)9	103	30623	792	2	5	497
2	7	85307	68	527	(35)7	5	51	8	529	9	4	61119	1(32)	1	426	20	872	6	348	611
3	090	(41)2	63	9	51955	8	5	817	78	113	672	431	2	6	73511	96	689	8(24)	7	4
4	825	96506	(98)4	6	97	705	5	632	858	45527	8	2	052	4	0	(16)2	23	1	601	92
5	(98)1	4	693	72723	33	88	069	3	20958	239	2(41)	2	125	1	7	8	8	06	389	534
6	65461	0	50	551	7(28)	394	3	11875	8	2	261	176	223	27	4	2(42)	82	6	328	7
7	10586	05	598	(16)9	4	7	54	839	6	08	9	5	1(73)	907	3	478	77364	038	2	976
8	8	742	91159	3(17)	73	395	4(39)	646	675	316	3	17	2	7	24	6	4	9	86011	780
9	5	384	49907	(08)3	05	1	676	9	2	70	4	827	7(72)	557	169	1	16317	5	18	652
10	9	(18)9	185	01	85453	8(32)	8	960	807	5	4	6	20777	57	4	68	879	446	623	7
11	(98)7	03297	0	94	215	6	39	495	039	(14)1	2	81975	742	8	967	3	9	7	87	436
12	12203	55	7	3(51)	355	245	9	728	880	129	9	15	47	77034	5	943	8	3(61)	6	8
13	6(12)	104	17912	7	94	03	3	6	2	883	9	316	299	43385	511	6	33	5	(62)8	704
14	153	43	(15)2	38738	9	96	879	1	307	0	85	9	5(54)	2	6	830	4	670	990	84797
15	9	76	86192	1(56)	359	13	6	83824	467	858	5	7	370	979	0	128	8	5(11)	56	5
16	(89)5	241	22400	6	18	9	95	7	60	238	817	7	946	(87)3	5	294	8	2	30067	129

Table S4. Additional information on behavioral performance: observed frequencies, durations, and proportions of correct solutions by trial type. The frequencies are given per experimental run (summed over the different distractor conditions), because in GLMs 3 and 4 (described in section 2.8.1), the trials were modeled across all distractor conditions, as there were no significant effects of distractor type on behavioral performance.

**Trial type**	**Number of trials per run (mean, range)**	**Duration of trials in seconds (mean, range)**	**Proportion correct (mean, range)**
Control (total)	39.2 (16-54.3)	6.1 (4.0-13.4)	99.8 % (98.7-100 %)
Solve (total)	44.2 (25.5-63.5)	5.2 (3.5-8.5)	97.7 % (93.4-100 %)
Solve simple	32.5 (17-49.3)	5.0 (3.3-8.4)	97.8 % (93.7-100 %)
Solve complex	11.7 (8.5-14.3)	5.8 (3.9-8.7)	97.3 % (87.9-100 %)
Create (total)	18.6 (14-26.5)	10.5 (6.7-14.3)	95.2 % (78.4-100 %)
Create simple	10.87 (6-15.3)	8.2 (5.5-12.7)	97.6 % (87.8-100 %)
Create complex	8.5 (2.3-16.5)	14.0 (8.8-19.0)	95.1 % (77.5-100 %)

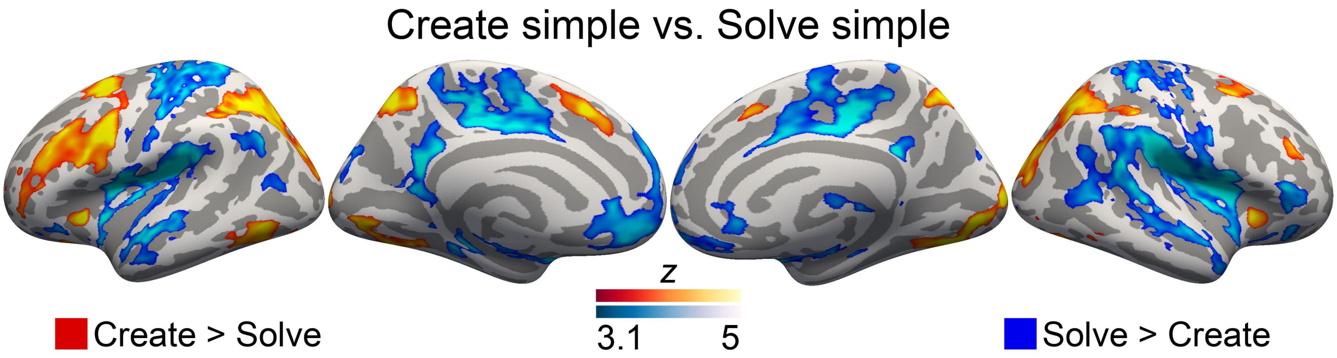

Figure S1*: Contrast between simple calculations in the create and solve tasks. Note that the regions activated more highly by the simple create calculations largely overlap with the regions where a parametric task-dependent modulation was observed in the analysis on the main effects of task (Fig. 3, main text).*

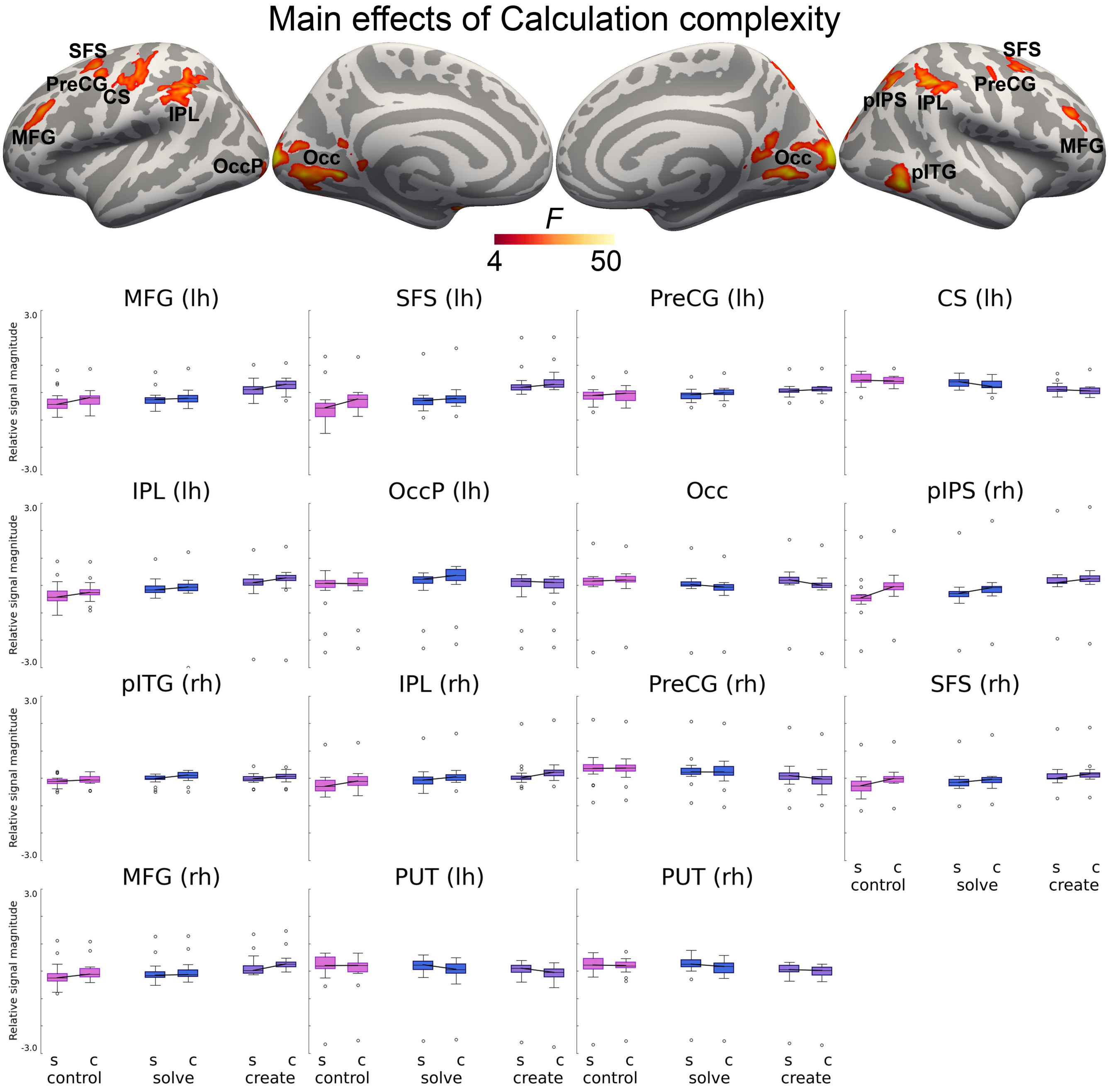

***Figure S2**: Main effects of Calculation complexity (initial cluster forming threshold P = 0.001, permuted cluster significance P < 0.05, FWER-corrected) from the 2 ×* 2 ANOVA *with factors task (solve, create) and calculation complexity (simple, complex). For ease of visual comparison, the mean signal magnitude across all ROIs and conditions was subtracted from all values. Abbreviations: MFG, middle frontal gyrus; SFS, superior frontal sulcus; PreCG, precentral gyrus; CS, central sulcus; IPL, inferior parietal lobule; OccP, Occipital pole; Occ, occipital; pIPS, posterior inferior parietal sulcus; pITG, posterior inferior temporal gyrus; PUT, putamen; lh, left hemisphere; rh, right hemisphere; s, simple, c, complex (in the control task, these refer to short and long expressions, respectively).*

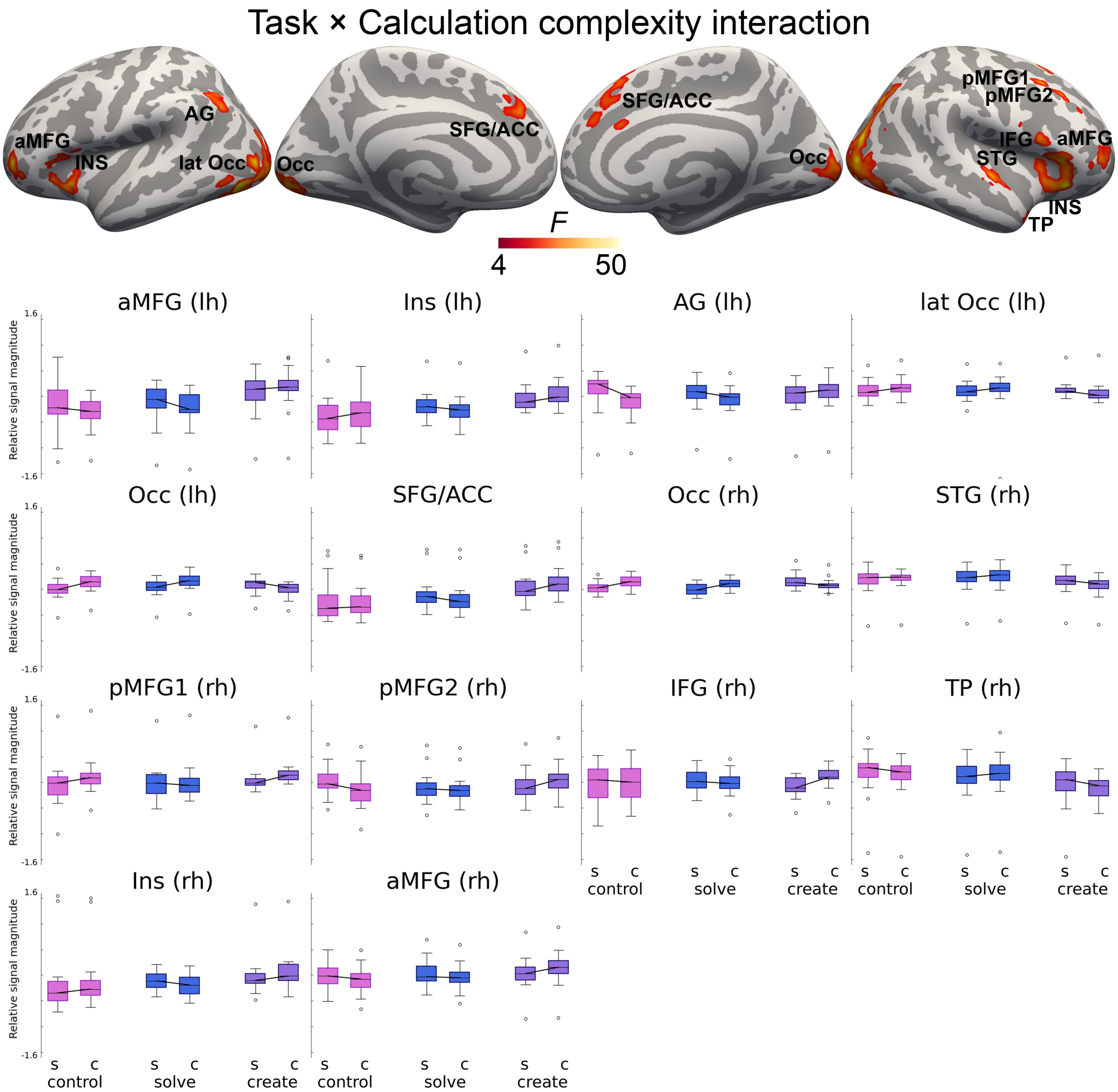

Figure S3*: Interactions between Task and Calculation complexity (initial cluster forming threshold P = 0.001, permuted cluster significance P < 0.05, FWER-corrected) from the 2 × 2 ANOVA with factors Task (solve, create) and calculation complexity (simple, complex). For ease of visual comparison, the mean signal magnitude across all clusters and conditions was subtracted from all values. Abbreviations: aMFG, anterior middle frontal gyrus; INS, insula; AG, angular gyrus; lat Occ, lateral occipital; Occ, occipital; SFG, superior frontal gyrus; ACC, anterior cingulate cortex; STG, superior temporal gyrus; pMFG, posterior middle frontal gyrus; IFG, inferior frontal gyrus; TP, temporal pole; lh, left hemisphere; rh, right hemisphere; s, simple, c, complex (in the control task, these refer to short and long expressions, respectively).*

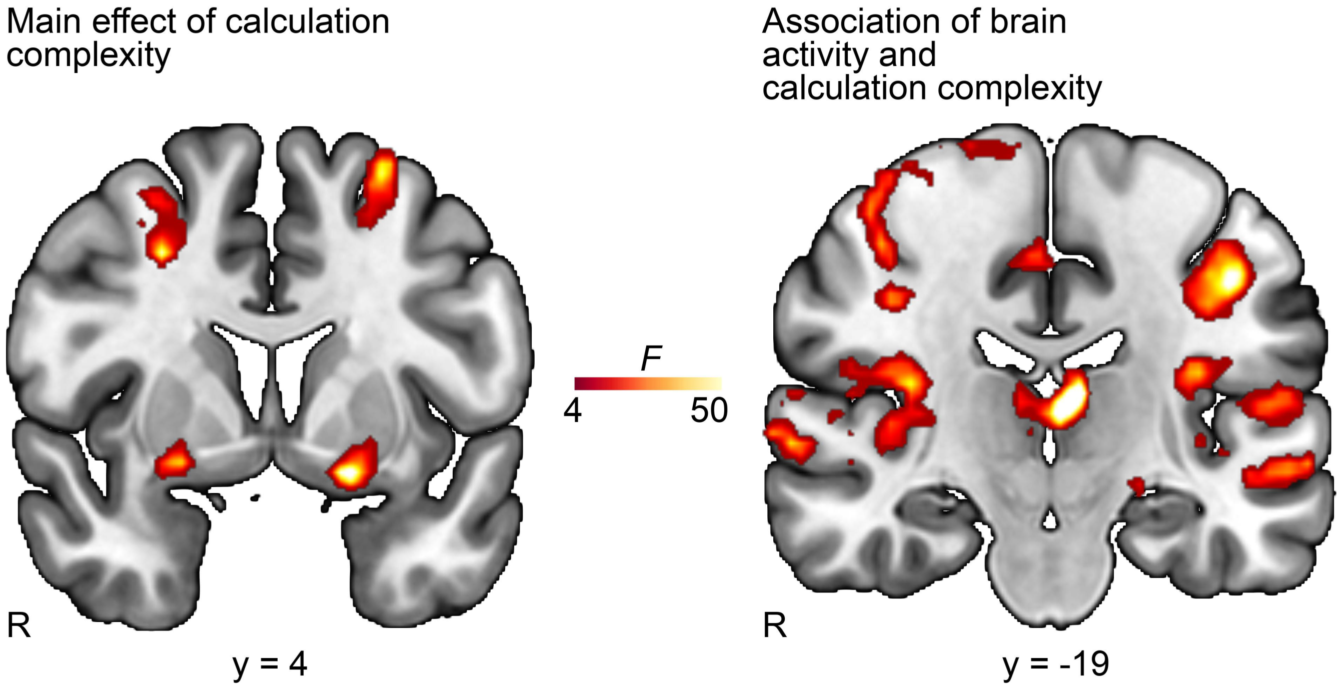

***Figure S4**: Left: a coronal slice from the analysis on main effects of Calculation complexity displaying clusters in the left and right putamen. Right: a coronal slice from the analysis on associations of brain activity and calculation complexity displaying a cluster in the thalamus.*

Table S5. Clusters observed in the analysis on the main effects of Task in the 3 × 4 ANOVA with factors Task (solve, create, control) and Distractor (speech, nonsense, vocoded, silence). Coordinates are given in MNI space.

Region (of peak activation coordinate)	# vox	Size (cm3)	x	y	z	Peak F-value
Left Insular Cortex	3780	59.06	-30	24	-2	208
Right Supramarginal Gyrus, posterior division	2955	46.17	65	-44	28	88.67
Left Precentral Gyrus	1146	17.91	-43	-14	61	91.73
Left Juxtapositional Lobule Cortex (formerly Supplementary Motor Cortex)	1121	17.52	0	-11	53	88.82
Left Accumbens	580	9.06	-3	7	-5	54
Right Crus I	568	8.88	35	-61	-30	97.68
Left Lateral Occipital Cortex, superior division	552	8.62	-35	-59	46	105.53
Left Precuneous Cortex	474	7.41	-5	-76	51	54.49
Right V	341	5.33	15	-51	-17	67.34
Right Occipital Pole	335	5.23	5	-94	-2	39.55
Right Angular Gyrus	331	5.17	40	-56	41	40.4
Right Paracingulate Gyrus	279	4.36	3	19	46	136.48
Left Middle Frontal Gyrus	263	4.11	-28	12	58	68.3
Right Cingulate Gyrus, posterior division	247	3.86	3	-46	31	53.71
Right Insular Cortex	241	3.77	33	24	1	88.16
Left VI	171	2.67	-30	-61	-32	73.48
Left Lateral Occipital Cortex, inferior division	157	2.45	-50	-66	-12	72.2
Right Crus I	155	2.42	13	-81	-22	76.99
Left Frontal Pole	155	2.42	0	57	16	51.86
Right Frontal Pole	141	2.2	48	39	33	47.89
Right VIIIa	135	2.11	25	-56	-52	42.02
Left Caudate	119	1.86	-15	-4	21	73.77
Right Cingulate Gyrus, anterior division	102	1.59	5	-1	31	89.8
Left Cingulate Gyrus, posterior division	77	1.2	-3	-31	26	43.96
Right I-IV	76	1.19	3	-49	-22	35.14
Left Precentral Gyrus	71	1.11	-58	-1	36	77.91
Right Superior Frontal Gyrus	68	1.06	28	12	63	39.2
Right Cingulate Gyrus, anterior division	67	1.05	10	34	18	26.75
Left Angular Gyrus	65	1.02	-43	-59	33	29.84
Left Thalamus	57	0.89	-8	-11	11	50.97
Right Occipital Pole	48	0.75	25	-99	-5	49.85
Right Postcentral Gyrus	48	0.75	33	-36	61	27.68
Left VI	47	0.73	-25	-54	-20	38.93
Right Inferior Frontal Gyrus, pars triangularis	44	0.69	55	34	11	36.93
Left VIIIa	42	0.66	-30	-54	-52	26.87
Left Crus II	40	0.62	-38	-61	-45	34.38

Table S6. Clusters observed in the analysis on the main effects of Distractor in the 3 × 4 ANOVA with factors Task (solve, create, control) and Distractor (speech, nonsense, vocoded, silence). Coordinates are given in MNI space.

Region (of peak activation coordinate)	# vox	Size (cm3)	x	y	z	Peak F-value
Left Planum Temporale	3049	47.64	-38	-29	11	166.87
Right Heschl's Gyrus (includes H1 and H2)	2191	34.23	50	-16	8	156.81
Left Lateral Occipital Cortex, superior division	519	8.11	-43	-59	53	50.32
Right Angular Gyrus	518	8.09	48	-46	56	23.44
Left Precuneous Cortex	487	7.61	-5	-71	43	40.25
Right Frontal Pole	325	5.08	43	47	18	22.9
Left Cingulate Gyrus, posterior division	288	4.5	-5	-31	28	39.44
Right Paracingulate Gyrus	281	4.39	3	34	26	45.37
Left Frontal Medial Cortex	238	3.72	0	44	-15	28.31
Left VI	102	1.59	-35	-46	-35	47.83
Right Crus II	99	1.55	23	-76	-37	28.74
Left Frontal Pole	99	1.55	-5	64	28	20.99
Right Thalamus	86	1.34	15	-26	-2	38.19
Left Superior Frontal Gyrus	79	1.23	-15	9	66	22.11
Left Frontal Pole	78	1.22	-8	47	48	20.4
Left Crus II	67	1.05	-45	-51	-52	18.87
Left Frontal Orbital Cortex	64	1	-50	29	-7	33.67
Left Frontal Pole	63	0.98	-45	39	11	21.41
Right VI	54	0.84	30	-54	-37	35.69
Left Middle Frontal Gyrus	51	0.8	-43	17	41	16.8
Left Frontal Pole	49	0.77	-30	59	26	21.81
Left Lateral Occipital Cortex, superior division	40	0.62	-30	-89	33	13.66
Right Inferior Temporal Gyrus, posterior division	37	0.58	55	-36	-20	21.44
Right Frontal Pole	35	0.55	30	49	-15	19.26

Table S7. Clusters observed in the analysis on the interaction between Task and Distractor in the 3 × 4 ANOVA with factors Task (solve, create, control) and Distractor (speech, nonsense, vocoded, silence). Coordinates are given in MNI space.

Region (of peak activation coordinate)	# vox	Size (cm3)	x	y	z	Peak F-value
Left Central Opercular Cortex	429	6.7	-60	-6	6	35.83
Right Superior Temporal Gyrus, posterior division	148	2.31	60	-24	1	23.44
Left Precuneous Cortex	34	0.53	-10	-69	41	14.81

Table S8. Clusters observed in the analysis on the main effects of Calculation complexity in the 2 × 2 ANOVA with factors Task (solve, create) and Calculation complexity (simple, complex). Coordinates are given in MNI space.

Region (of peak activation coordinate)	# vox	Size (cm3)	x	y	z	Peak F-value
Right Occipital Pole	1497	23.39	8	-94	11	139.23
Left Supramarginal Gyrus, posterior division	461	7.2	-40	-46	43	76.14
Right Supramarginal Gyrus, posterior division	438	6.84	40	-41	43	62.06
Right Lateral Occipital Cortex, superior division	287	4.48	30	-64	48	50.05
Left Postcentral Gyrus	190	2.97	-43	-19	38	56.72
Right Middle Temporal Gyrus, temporooccipital part	136	2.12	58	-59	-7	71.06
Left Middle Frontal Gyrus	111	1.73	-28	4	63	45.77
Right Middle Frontal Gyrus	110	1.72	28	4	43	50.18
Left Putamen	84	1.31	-20	7	-12	63.43
Right Middle Frontal Gyrus	78	1.22	43	34	31	31.91
Left Middle Frontal Gyrus	76	1.19	-43	34	41	41.49
Left Middle Frontal Gyrus	52	0.81	-33	-1	41	86.44
Right Putamen	48	0.75	23	4	-10	39.16
Right Precentral Gyrus	45	0.7	55	-9	51	31.55
Left Occipital Pole	38	0.59	-25	-91	-5	30.94

Table S9. Clusters observed in the analysis on the interaction between Task and Calculation complexity in the 2 × 2 ANOVA with factors Task (solve, create) and Calculation complexity (simple, complex). Coordinates are given in MNI space.

Region (of peak activation coordinate)	# vox	Size (cm3)	x	y	z	Peak F-value
Right Lateral Occipital Cortex, superior division	841	13.14	38	-86	13	72.83
Left Lateral Occipital Cortex, inferior division	543	8.48	-43	-71	-10	95.98
Right Frontal Orbital Cortex	258	4.03	43	24	-10	78.1
Right Superior Frontal Gyrus	223	3.48	3	29	56	41.66
Left Frontal Orbital Cortex	171	2.67	-35	22	-7	56.8
Right Frontal Pole	135	2.11	43	47	6	32.32
Left Lateral Occipital Cortex, superior division	117	1.83	-43	-64	36	32.82
Left Frontal Pole	96	1.5	-38	54	3	64.99
Right Planum Temporale	81	1.27	60	-9	1	39.73
Right Inferior Frontal Gyrus, pars opercularis	66	1.03	48	9	16	31.69
Right Frontal Pole	55	0.86	35	39	41	39.75
Right Temporal Pole	48	0.75	40	12	-30	35.44
Right Middle Frontal Gyrus	46	0.72	38	17	53	43.27
Left Lateral Occipital Cortex, inferior division	43	0.67	-43	-84	6	51.34

Table S10. Clusters observed in the analysis on the associations between brain activity and calculation complexity. Coordinates are given in MNI space.

Region (of peak activation coordinate)	# vox	Size (cm3)	x	y	z	Peak F-value
Left Lingual Gyrus	4446	69.47	-8	-86	-15	157.57
Left Frontal Pole	4147	64.8	-38	59	8	90.78
Right Planum Polare	2592	40.5	53	-1	1	122.3
Left Central Opercular Cortex	948	14.81	-60	-14	11	90.43
Left Supramarginal Gyrus, posterior division	592	9.25	-50	-49	51	54.58
Right Supramarginal Gyrus, posterior division	576	9	53	-36	53	83.31
Left Crus II	487	7.61	-28	-76	-52	81.97
Right Crus II	449	7.02	35	-64	-42	60.99
Left Inferior Temporal Gyrus, temporooccipital part	253	3.95	-58	-61	-10	61.64
Right Cingulate Gyrus, anterior division	208	3.25	10	-11	41	37.24
Left Postcentral Gyrus	206	3.22	-45	-19	41	63.79
Left Thalamus	197	3.08	-5	-19	11	92.86
Right Middle Temporal Gyrus, temporooccipital part	143	2.23	58	-54	-7	56.04
Right Lateral Occipital Cortex, superior division	108	1.69	20	-61	58	64.93
Left Amygdala	108	1.69	-20	-1	-15	31.62
Right Precentral Gyrus	104	1.62	53	9	26	42.58
Left Lateral Ventricle	101	1.58	-25	-54	11	39.55
Right Temporal Pole	80	1.25	40	19	-25	56.32
Left Superior Parietal Lobule	77	1.2	-30	-56	56	36.4
Right Lateral Ventricle	52	0.81	23	-39	21	31.15
Left Frontal Orbital Cortex	51	0.8	-30	17	-27	46.45
Right Angular Gyrus	48	0.75	58	-49	28	33.26
Right Thalamus	37	0.58	15	-29	-2	51.15
Right VIIb	36	0.56	8	-71	-42	88.48
Left Amygdala	35	0.55	-23	-16	-12	33.37
Left Cerebral White Matter	34	0.53	-20	4	26	31.41

Preprocessing of (f)MRI data

Results included in this manuscript come from preprocessing performed using *fMRIPrep* 20.2.5 (Esteban, Markiewicz, et al. (2018); Esteban, Blair, et al. (2018); RRID:SCR_016216), which is based on *Nipype* 1.6.1 (Gorgolewski et al. (2011); Gorgolewski et al. (2018); RRID:SCR_002502).

Anatomical data preprocessing

> A total of 1 T1-weighted (T1w) images were found within the input BIDS dataset.The T1-weighted (T1w) image was corrected for intensity non-uniformity (INU) with `N4BiasFieldCorrection` (Tustison et al. 2010), distributed with ANTs 2.3.3 (Avants et al. 2008, RRID:SCR_004757), and used as T1w-reference throughout the workflow. The T1w-reference was then skull-stripped with a *Nipype* implementation of the `antsBrainExtraction.sh` workflow (from ANTs), using OASIS30ANTs as target template. Brain tissue segmentation of cerebrospinal fluid (CSF), white-matter (WM) and gray-matter (GM) was performed on the brain-extracted T1w using `fast` (FSL 5.0.9, RRID:SCR_002823, Zhang, Brady, and Smith 2001). Brain surfaces were reconstructed using `recon-all` (FreeSurfer 6.0.1, RRID:SCR_001847, Dale, Fischl, and Sereno 1999), and the brain mask estimated previously was refined with a custom variation of the method to reconcile ANTs-derived and FreeSurfer-derived segmentations of the cortical gray-matter of Mindboggle (RRID:SCR_002438, Klein et al. 2017). Volume-based spatial normalization to one standard space (MNI152NLin6Asym) was performed through nonlinear registration with `antsRegistration` (ANTs 2.3.3), using brain-extracted versions of both T1w reference and the T1w template. The following templates were selected for spatial normalization: *FSL's MNI ICBM 152 non-linear 6th Generation Asymmetric Average Brain Stereotaxic Registration Model* [Evans et al. (2012), RRID:SCR_002823; TemplateFlow ID: MNI152NLin6Asym].

Functional data preprocessing

> For each of the 4 BOLD runs found per subject (across all tasks and sessions), the following preprocessing was performed. First, a reference volume and its skull-stripped version were generated using a custom methodology of *fMRIPrep*. A deformation field to correct for susceptibility distortions was estimated based on *fMRIPrep*'s *fieldmap-less* approach. The deformation field is that resulting from co-registering the BOLD reference to the same-subject T1w-reference with its intensity inverted (Wang et al. 2017; Huntenburg 2014). Registration is performed with `antsRegistration` (ANTs 2.3.3), and the process regularized by constraining deformation to be nonzero only along the phase-encoding direction, and modulated with an average fieldmap template (Treiber et al. 2016). Based on the estimated susceptibility distortion, a corrected EPI (echo-planar imaging) reference was calculated for a more accurate co-registration with the anatomical reference. The BOLD reference was then co-registered to the T1w reference using `bbregister` (FreeSurfer) which implements boundary-based registration (Greve and Fischl 2009). Co-registration was configured with six degrees of freedom. Head-motion parameters with respect to the BOLD reference (transformation matrices, and six corresponding rotation and translation parameters) are estimated before any spatiotemporal filtering using `mcflirt` (FSL 5.0.9, Jenkinson et al. 2002). BOLD runs were slice-time corrected to 0.594s (0.5 of slice acquisition range 0s-1.19s) using `3dTshift` from AFNI

20160207 (Cox and Hyde 1997, RRID:SCR_005927). The BOLD time-series were resampled onto the following surfaces (FreeSurfer reconstruction nomenclature): *fsaverage*, *fsnative*. The BOLD time-series (including slice-timing correction when applied) were resampled onto their original, native space by applying a single, composite transform to correct for head-motion and susceptibility distortions. These resampled BOLD time-series will be referred to as *preprocessed BOLD in original space*, or just *preprocessed BOLD*. The BOLD time-series were resampled into standard space, generating a *preprocessed BOLD run in MNI152NLin6Asym space*. First, a reference volume and its skull-stripped version were generated using a custom methodology of *fMRIPrep*. Several confounding time-series were calculated based on the *preprocessed BOLD*: framewise displacement (FD), DVARS and three region-wise global signals. FD was computed using two formulations following Power (absolute sum of relative motions, Power et al. (2014)) and Jenkinson (relative root mean square displacement between affines, Jenkinson et al. (2002)). FD and DVARS are calculated for each functional run, both using their implementations in *Nipype* (following the definitions by Power et al. 2014). The three global signals are extracted within the CSF, the WM, and the whole-brain masks. Additionally, a set of physiological regressors were extracted to allow for component-based noise correction (*CompCor*, Behzadi et al. 2007). Principal components are estimated after high-pass filtering the *preprocessed BOLD* time-series (using a discrete cosine filter with 128s cut-off) for the two *CompCor* variants: temporal (tCompCor) and anatomical (aCompCor). tCompCor components are then calculated from the top 2% variable voxels within the brain mask. For aCompCor, three probabilistic masks (CSF, WM and combined CSF+WM) are generated in anatomical space. The implementation differs from that of Behzadi et al. in that instead of eroding the masks by 2 pixels on BOLD space, the aCompCor masks are subtracted a mask of pixels that likely contain a volume fraction of GM. This mask is obtained by dilating a GM mask extracted from the FreeSurfer's *aseg* segmentation, and it ensures components are not extracted from voxels containing a minimal fraction of GM. Finally, these masks are resampled into BOLD space and binarized by thresholding at 0.99 (as in the original implementation). Components are also calculated separately within the WM and CSF masks. For each CompCor decomposition, the *k* components with the largest singular values are retained, such that the retained components' time series are sufficient to explain 50 percent of variance across the nuisance mask (CSF, WM, combined, or temporal). The remaining components are dropped from consideration. The head-motion estimates calculated in the correction step were also placed within the corresponding confounds file. The confound time series derived from head motion estimates and global signals were expanded with the inclusion of temporal derivatives and quadratic terms for each (Satterthwaite et al. 2013). Frames that exceeded a threshold of 0.5 mm FD or 1.5 standardised DVARS were annotated as motion outliers. All resamplings can be performed with *a single interpolation step* by composing all the pertinent transformations (i.e. head-motion transform matrices, susceptibility distortion correction when available, and co-registrations to anatomical and output spaces). Gridded (volumetric) resamplings were performed using `antsApplyTransforms` (ANTs), configured with Lanczos interpolation to minimize the smoothing effects of other kernels (Lanczos 1964). Non-gridded (surface) resamplings were performed using `mri_vol2surf` (FreeSurfer).

Many internal operations of *fMRIPrep* use *Nilearn* 0.6.2 (Abraham et al. 2014, RRID:SCR_001362), mostly within the functional processing workflow. For more details of the pipeline, see [the section corresponding to workflows in *fMRIPrep*'s documentation].

Copyright Waiver

The above boilerplate text was automatically generated by fMRIPrep with the express intention that users should copy and paste this text into their manuscripts *unchanged*. It is released under the CC0 license.

References

Abraham, Alexandre, Fabian Pedregosa, Michael Eickenberg, Philippe Gervais, Andreas Mueller, Jean Kossaifi, Alexandre Gramfort, Bertrand Thirion, and Gael Varoquaux. 2014. "Machine Learning for Neuroimaging with Scikit-Learn." *Frontiers in Neuroinformatics* 8. https://doi.org/10.3389/fninf.2014.00014.

Avants, B.B., C.L. Epstein, M. Grossman, and J.C. Gee. 2008. "Symmetric Diffeomorphic Image Registration with Cross-Correlation: Evaluating Automated Labeling of Elderly and Neurodegenerative Brain." *Medical Image Analysis* 12 (1): 26–41. https://doi.org/10.1016/j.media.2007.06.004.

Behzadi, Yashar, Khaled Restom, Joy Liau, and Thomas T. Liu. 2007. "A Component Based Noise Correction Method (CompCor) for BOLD and Perfusion Based fMRI." *NeuroImage* 37 (1): 90–101. https://doi.org/10.1016/j.neuroimage.2007.04.042.

Cox, Robert W., and James S. Hyde. 1997. "Software Tools for Analysis and Visualization of fMRI Data." *NMR in Biomedicine* 10 (4-5): 171–78. https://doi.org/10.1002/(SICI)1099-1492(199706/08)10:4/5<171::AID-NBM453>3.0.CO;2-L.

Dale, Anders M., Bruce Fischl, and Martin I. Sereno. 1999. "Cortical Surface-Based Analysis: I. Segmentation and Surface Reconstruction." *NeuroImage* 9 (2): 179–94. https://doi.org/10.1006/nimg.1998.0395.

Esteban, Oscar, Ross Blair, Christopher J. Markiewicz, Shoshana L. Berleant, Craig Moodie, Feilong Ma, Ayse Ilkay Isik, et al. 2018. "FMRIPrep." *Software*. Zenodo. https://doi.org/10.5281/zenodo.852659.

Esteban, Oscar, Christopher Markiewicz, Ross W Blair, Craig Moodie, Ayse Ilkay Isik, Asier Erramuzpe Aliaga, James Kent, et al. 2018. "fMRIPrep: A Robust Preprocessing Pipeline for Functional MRI." *Nature Methods*. https://doi.org/10.1038/s41592-018-0235-4.

Evans, AC, AL Janke, DL Collins, and S Baillet. 2012. "Brain Templates and Atlases." *NeuroImage* 62 (2): 911–22. https://doi.org/10.1016/j.neuroimage.2012.01.024.

Fonov, VS, AC Evans, RC McKinstry, CR Almli, and DL Collins. 2009. "Unbiased Nonlinear Average Age-Appropriate Brain Templates from Birth to Adulthood." *NeuroImage* 47, Supplement 1: S102. https://doi.org/10.1016/S1053-8119(09)70884-5.

Gorgolewski, K., C. D. Burns, C. Madison, D. Clark, Y. O. Halchenko, M. L. Waskom, and S. Ghosh. 2011. "Nipype: A Flexible, Lightweight and Extensible Neuroimaging Data Processing Framework in Python." *Frontiers in Neuroinformatics* 5: 13. https://doi.org/10.3389/fninf.2011.00013.

Gorgolewski, Krzysztof J., Oscar Esteban, Christopher J. Markiewicz, Erik Ziegler, David Gage Ellis, Michael Philipp Notter, Dorota Jarecka, et al. 2018. “Nipype.” *Software*. Zenodo. https://doi.org/10.5281/zenodo.596855.

Greve, Douglas N, and Bruce Fischl. 2009. “Accurate and Robust Brain Image Alignment Using Boundary-Based Registration.” *NeuroImage* 48 (1): 63–72. https://doi.org/10.1016/j.neuroimage.2009.06.060.

Huntenburg, Julia M. 2014. “Evaluating Nonlinear Coregistration of BOLD EPI and T1w Images.” Master’s Thesis, Berlin: Freie Universität. http://hdl.handle.net/11858/00-001M-0000-002B-1CB5-A.

Jenkinson, Mark, Peter Bannister, Michael Brady, and Stephen Smith. 2002. “Improved Optimization for the Robust and Accurate Linear Registration and Motion Correction of Brain Images.” *NeuroImage* 17 (2): 825–41. https://doi.org/10.1006/nimg.2002.1132.

Klein, Arno, Satrajit S. Ghosh, Forrest S. Bao, Joachim Giard, Yrjö Häme, Eliezer Stavsky, Noah Lee, et al. 2017. “Mindboggling Morphometry of Human Brains.” *PLOS Computational Biology* 13 (2): e1005350. https://doi.org/10.1371/journal.pcbi.1005350.

Lanczos, C. 1964. “Evaluation of Noisy Data.” *Journal of the Society for Industrial and Applied Mathematics Series B Numerical Analysis* 1 (1): 76–85. https://doi.org/10.1137/0701007.

Power, Jonathan D., Anish Mitra, Timothy O. Laumann, Abraham Z. Snyder, Bradley L. Schlaggar, and Steven E. Petersen. 2014. “Methods to Detect, Characterize, and Remove Motion Artifact in Resting State fMRI.” *NeuroImage* 84 (Supplement C): 320–41. https://doi.org/10.1016/j.neuroimage.2013.08.048.

Satterthwaite, Theodore D., Mark A. Elliott, Raphael T. Gerraty, Kosha Ruparel, James Loughead, Monica E. Calkins, Simon B. Eickhoff, et al. 2013. “An improved framework for confound regression and filtering for control of motion artifact in the preprocessing of resting-state functional connectivity data.” *NeuroImage* 64 (1): 240–56. https://doi.org/10.1016/j.neuroimage.2012.08.052.

Treiber, Jeffrey Mark, Nathan S. White, Tyler Christian Steed, Hauke Bartsch, Dominic Holland, Nikdokht Farid, Carrie R. McDonald, Bob S. Carter, Anders Martin Dale, and Clark C. Chen. 2016. “Characterization and Correction of Geometric Distortions in 814 Diffusion Weighted Images.” *PLOS ONE* 11 (3): e0152472. https://doi.org/10.1371/journal.pone.0152472.

Tustison, N. J., B. B. Avants, P. A. Cook, Y. Zheng, A. Egan, P. A. Yushkevich, and J. C. Gee. 2010. “N4ITK: Improved N3 Bias Correction.” *IEEE Transactions on Medical Imaging* 29 (6): 1310–20. https://doi.org/10.1109/TMI.2010.2046908.

Wang, Sijia, Daniel J. Peterson, J. C. Gatenby, Wenbin Li, Thomas J. Grabowski, and Tara M. Madhyastha. 2017. “Evaluation of Field Map and Nonlinear Registration Methods for Correction of Susceptibility Artifacts in Diffusion MRI.” *Frontiers in Neuroinformatics* 11. https://doi.org/10.3389/fninf.2017.00017.

Zhang, Y., M. Brady, and S. Smith. 2001. “Segmentation of Brain MR Images Through a Hidden Markov Random Field Model and the Expectation-Maximization Algorithm.” *IEEE Transactions on Medical Imaging* 20 (1): 45–57. https://doi.org/10.1109/42.906424.
